# The Underrepresentation of Females in Studies Assessing the Impact of High-Dose Exercise on Cardiovascular Outcomes: a Scoping Review

**DOI:** 10.1186/s40798-021-00320-y

**Published:** 2021-04-29

**Authors:** Roshan Patel, Caitlin L. Kemp, Muneebah Hafejee, Nicholas Peckham, Vageesh Jain, Gerry P. McCann, Susil Pallikadavath

**Affiliations:** 1 College of Life Sciences, University of Leicester, Leicester, UK; 2 Hereford County Hospital, Hereford, UK; 3 Centre for Statistics in Medicine, University of Oxford, Oxford, UK; 4 Institute for Global Health, University College London, London, UK; 5 NIHR Leicester Biomedical Research Centre for Cardiovascular Disease, Glenfield Hospital, University of Leicester, Leicester, UK

**Keywords:** Exercise, Female participants, Athletes, Cardiovascular outcomes

## Abstract

High-dose exercise-induced cardiac outcomes may vary between sexes. However, many studies investigating the cardiovascular effects of high-dose exercise have excluded or under-recruited females. This scoping review aimed to describe the recruitment of females in studies assessing the impact of high-dose exercise on cardiovascular outcomes and describe how this has changed over time. This scoping review followed the protocol outlined by Arksey and O’Malley and is reported as per the Preferred Reporting Items for Systematic Reviews and Meta-Analyses extension for scoping reviews (PRISMA-ScR) guidelines. The OVID and EMBASE databases were searched for studies that assessed the effects of high-dose exercise on cardiovascular outcomes. Both professional and nonprofessional groups were included. The review found 2973 studies, and 250 met the inclusion criteria including cumulatively 17,548,843 subjects. Over half the studies (*n* = 127) excluded females entirely, and only 8 (3.2%) studies recruited all-female participants. The overall mean percentage of females recruited was 18.2%. The mean percentage was 14.5% in studies conducted before 2011 and 21.8% in studies conducted after 2011. Females are an underrepresented group in studies assessing the cardiovascular outcomes related to high-dose exercise. As cardiovascular outcomes vary between sexes, translating findings from a largely male-based evidence may not be appropriate. Future investigators should aim to establish and overcome barriers to female recruitment.

**Supplementary Information:**

The online version contains supplementary material available at 10.1186/s40798-021-00320-y.

## Key Points

- Exercise-induced cardiac adaptation varies between males and females.
- Females may be underrepresented in the literature assessing the impacts of high-dose exercise on cardiovascular outcomes.
- Studies must focus on fair recruitment of both sexes such that policymakers can make decisions based on evidence for each sex rather than extrapolating from male-dominant data.

## Introduction

Exercise is an umbrella term that can be used to describe a variety of physical activity participation. The wide range of exercise types makes classification and dosing difficult, but this is clinically accomplished by utilising the Frequency, Intensity, Time and Type (FITT) principle [1]. This approach can loosely divide participants into low, middle and high doses. While exercise performed at high doses is classically associated with professional athletes, there are a large number of nonprofessionals who participate in exercise to an extent that would be classified as high dose [2].

While the benefits of exercise for cardiovascular health are well reported [3], the adverse cardiovascular effects of high-dose exercise are less clear [4]. Cardiac adaptation as a result of long-term, frequent and intense exercise is often termed the “athlete’s heart” [5]. Changes seen include bradycardia, ventricular and atrial dilatation and increased muscle thickness [5]. While these changes are well described in the scientific literature, the prognostic implications and hard cardiovascular end points are less well known. The strongest evidence for adverse clinical outcomes as a result of high-dose exercise is the increased risk of developing cardiac arrhythmias in later life [6–8]. For example, Andersen et al. found that race participation amongst cross-country skiers was associated with an increased risk of any arrhythmia [9] (hazard ratio (HR) = 1.30). The strongest associations were found with atrial fibrillation (AF) (HR = 1.29) and bradyarrhythmias (HR = 2.10). There is early evidence of a higher prevalence of coronary artery calcification (CAC) in endurance athletes when compared with non-athletic individuals and this may be related to total exercise dose [10]. However, causality, pathophysiology and prognostic implications of CAC in athletic groups is uncertain, particularly as plaques in this group may be a benign adaptation and do not carry the same prognostic implication as seen in the general population [10–13]. Myocardial fibrosis observed on cardiac magnetic resonance (CMR) represents another aspect of the athlete’s heart that represents uncertain pathophysiology, prognosis and clinical implications [14]. Fibrosis in athletes may be a result of the same pathophysiological, ischaemic changes more commonly observed in the general population [14, 15]. However, as argued by Baggish, fibrosis in athletes may portray a “two-hit process” where myocardial injury by infection and subclinical myocarditis is then aggravated by endurance exercise leading to more lasting changes such as fibrosis [15].

As the absolute number of professionals and nonprofessionals engaging in high-dose exercise rises [4], establishing the cardiovascular risks comes into focus. However, it is important that future studies represent participant groups fairly. Sex is one example [16, 17] that has gained focus as female participation in high-dose exercise is rising; it has increased by almost 75% in Olympic events over the last 30 years [18].

Cardiovascular risks are not homogenous between males and females. For example, the risks and presentation of ischaemic heart disease vary between sexes [19]. There is evidence to suggest female cardiac adaptations may differ from those seen in their male counterparts. For example, females may be more likely to undergo concentric remodelling [20, 21]. Studies that have demonstrated myocardial fibrosis have observed these adaptions only in their male participants [11, 22]. This sex-related variation in cardiovascular risk may also translate into risks associated with high-dose exercise [23].

Females may be underrepresented in studies assessing the relationship between high-dose exercise and cardiovascular outcomes [24–26]. While individual studies may have reason to recruit male-only participants, the overall literature base should reflect fair representation. It is important that the current representation of females in this literature base is evaluated, so that future research can target potentially underrepresented groups. This scoping review aims to describe the recruitment of females in the literature evaluating the effects of high-dose exercise on cardiovascular outcomes. It will analyse the proportion of studies excluding females and assess the mean percentage of females recruited within studies. Then, it will consider how recruitment trends have changed over time and by region.

## Methods

This scoping review was conducted and reported in accordance with the Preferred Reporting Items for Systematic Reviews and Meta-Analyses extension for scoping reviews (PRISMA-ScR) guidelines [27] and the methodological framework outlined by Arksey and O’Malley [28]. The five stages are detailed below. This review protocol was preregistered with Open Science Framework (author anonymised link: https://osf.io/dujpb/?view_only=00b65bc898ad4815a45cd1cb7c2d12ec).

### Stage 1: Identifying the Research Question

This review aimed to assess the level of female recruitment in studies evaluating the impact of high-dose exercise on cardiovascular outcomes. Our key research questions were

1. How many studies recruit female participants?
2. What percentage of female participants are recruited in studies?
3. Has the recruitment of female participants changed over time?

### Stage 2: Identify Relevant Studies

Key search terms were selected using a Population Intervention Control Outcome (PICO) framework to identify studies pertinent to our research aim (Supplement 1). Both MeSH/subject headings and title abstract search terms were included as appropriate. The full search strategy used for both OVID and EMBASE is outlined in Supplement 2. The dates included were from July 1946 to 24 June 2020. The search was performed on MEDLINE and EMBASE on 24 June 2020.

### Stage 3: Study Selection

**Fig. 1:**
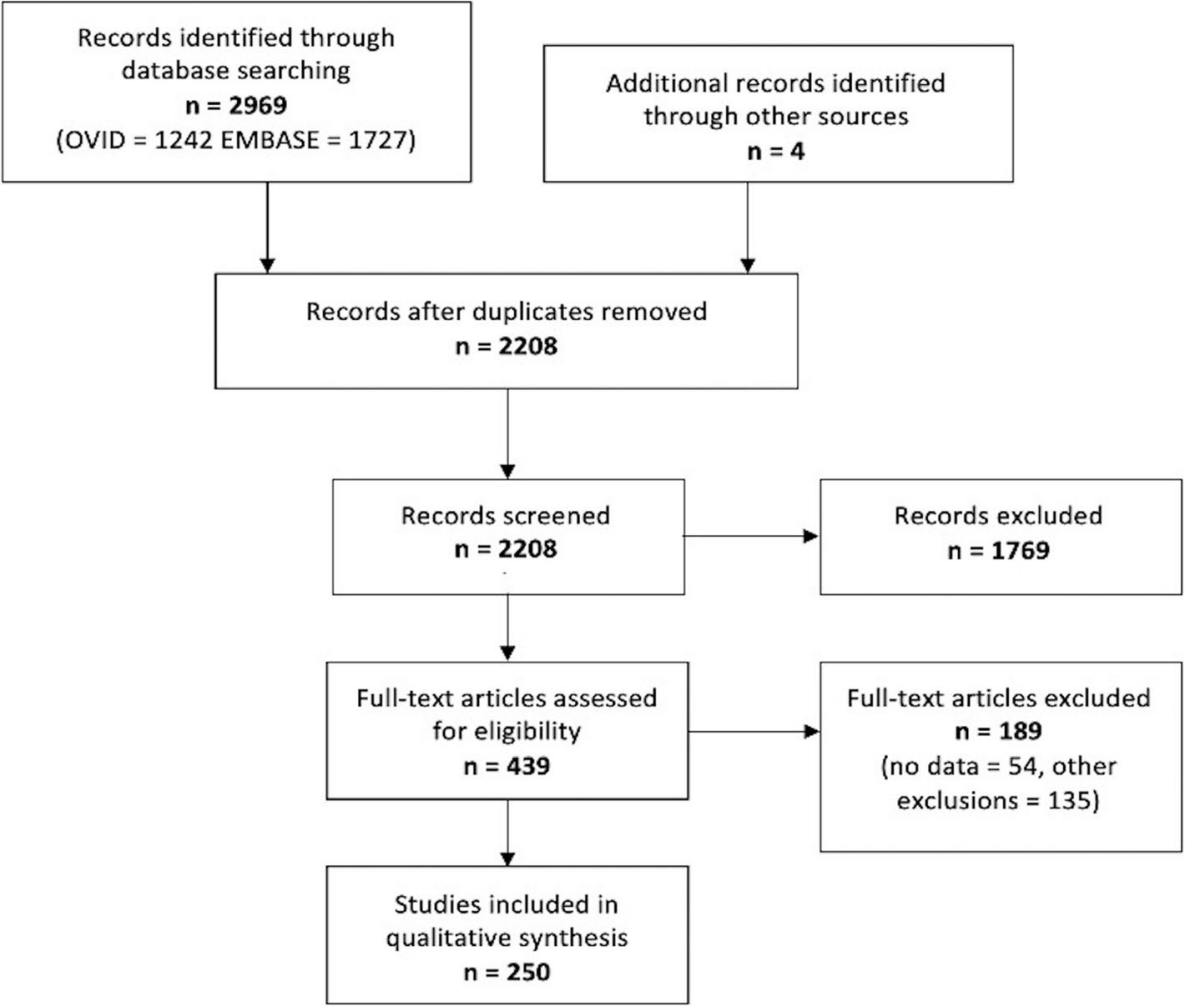
Preferred Reporting Items for Systematic Reviews and Meta-Analyses (PRISMA) flow diagram showing study exclusions

This review included all observational (prospective or retrospective cohort, case control or cross-sectional studies) or experimental studies that were published in English. Studies must have measured a cardiovascular outcome (arrhythmia, cardiomyopathy, ischaemic heart disease or other) or associated clinical marker, and the subjects must have participated in high-dose exercise, as defined by the respective investigators, to be included. As high-dose exercise is poorly defined, only studies which specified high-dose exercise participation or assessed activities widely accepted as high dose were included in analysis. These were marathon running, triathlon, endurance cycling, endurance rowing, endurance kayaking, ultra-distance events and cross-country skiing. The review included high-dose exercise in all populations (athlete and non-athlete individuals). Titles, abstracts and full texts were all independently reviewed by two authors (two of RP, CK, MH, SP). Any disagreements were discussed and the paper re-reviewed. If authors still disagreed following discussion, a third author was consulted who did not initially screen the paper (RP or SP). Studies retrieved through other sources, such as references within included studies, are shown in Fig. 1 (*n* = 4). Fifty-four studies were excluded due to lack of data or an inability to gain access to the full text (Fig. 1). Studies were also excluded if

- It did not meet the inclusion criteria.
- It was not published in English.
- Required data were missing.
- It was a case report, review article, conference related paper, protocol or editorial.

### Stage 4: Charting the Data

The data were extracted and entered into a spreadsheet programme (Microsoft Excel for Mac Version 16.39). The authors extracted the following information from the studies:

- Author name and year of publication
- The total number of participants
- Inclusion of female participants (yes/no)
- The total number of female participants
- Characteristic of the outcome of interest: clinical marker or a hard-clinical endpoint. A clinical endpoint was determined by diagnosis of a cardiovascular condition (categorised into arrhythmia, cardiomyopathy, ischaemic heart disease or other). A clinical marker was considered to be any indication that suggests an increased risk of cardiac pathology. Examples of clinical markers included imaging-based outcomes, subclinical electrocardiogram markers and haematological markers (e.g. troponin).
- The type of exercise assessed: running, triathlon, cross-country skiing, multiple or other. These categories were chosen from all of the exercises in the search results. The other category is an amalgamation of the remaining activities with an individual frequency that was deemed too low for a distinct category.
- The location of the study divided into World Health Organisation (WHO) Regions: African Region, Region of the Americas, South-East Asia Region, European Region, Eastern Mediterranean Region and the Western Pacific Region [29]

### Stage 5: collating, summarizing and reporting the results

A “descriptive–analytical” method was utilised to summarise and report key findings pertinent to the research question for each study. Studies were further organised to aid assessment of temporal trends and the influence of factors which may have affected recruitment of participants (study location and exercise type).

Studies were categorised into groups according to when they were published, whether they assessed clinical markers or endpoints, the WHO region they were conducted within and the exercise type assessed. Temporal trends were analysed using two methods: (1) Studies were split into two equal groups around the median year of publication (2011) so that gross trends in female recruitment can be compared in the two halves of the literature. (2) Studies were divided into ten equal groups according to the time of publication so that trends can be seen over time.

### Descriptive Analysis

This study used two key calculations when analysing recruitment of females; percentage of studies excluding females (aim 1) and mean percentage of females recruited (aim 2). These were calculated for the dataset as a whole and for each of the following variables: temporal groups, WHO regions and exercise types. The mean percentage takes each study to be the base unit for the analysis. This measure was chosen for analysis instead of raw female participant data in order to account for the influence of larger studies.

## Results

### Overview

The search returned 2969 results (Fig. 1). Duplicate titles were removed (*n* = 765), and 2208 studies were reviewed. Two hundred and fifty studies (Supplementary document 4 and 5) were included in the analysis. Plotting the total population of each study (*n* = 17,548,843) revealed a non-normal negatively skewed distribution. The median study size was 73 (interquartile range (IQR): 33–246.5). The study publication dates ranged from 1964 to 2020, with 2011 as the year that the median number of studies was reported (IQR: 2000–2016). Of the 250 studies, 171 (68.4%) assessed clinical markers, and only 77 assessed cardiovascular clinical outcomes. Of the studies assessing cardiovascular outcomes, 30 (39.0%) assessed arrhythmias compared with 11 (14.3%) and 10 (13%) assessing cardiomyopathies and ischaemic heart disease. Twenty-six studies (33.8%) assessed “other” clinical outcomes which included hypertension, sudden cardiac death and cardiovascular mortality.

Over half the studies in this dataset did not recruit females (*n* = 127, 50.8%, Table 1), and only eight studies included all-female participants. The mean percentage of females recruited was 18.2%. The total percentage of females recruited overall was 43.1%. This was calculated by dividing the total number of females recruited in all studies (*n* = 7,556,552) by the total number of participants in all studies (*n* = 17,548,843). This was significantly influenced by larger, population-based studies. The top ten largest studies account for 99.2% of the total population (details are given in Table 2). While using ‘the top ten’ largest studies is arbitrary, this has been mainly used to illustrate the influence of these large studies. For clarity, supplement 3 outlines how the percentage of total females recruited varies by the number of studies excluded (largest first). When the top ten largest studies were excluded, the raw percentage (18.8%) and mean percentage (18.2%) values were similar.

**Table 1:** Female recruitment characteristics: changes pre vs post 2011 and overall. Large studies excluded and their characteristics are given in Table 2. Median number of participants in the excluded studies: 548,092 (IQR: 133,450–2,227,219). Range: 46,907–10,871,000

	Mean percentage females recruited per study (%)	Percentage of females recruited excluding large studies (%)	Percentage studies excluding females (%)
Overall (*n* = 250)	18.2	18.8	50.8 (*n* = 127)
Studies before 2011 (*n* = 121)	14.5	11.1	59.5 (*n* = 72)
Studies after 2011 (*n* = 129)	21.8	23.7	42.6 (*n* = 55)

**Table 2:** Characteristics of the large studies excluded from total percentage recruitment *Calculation*

Studies excluded	Number of participants	Percentage of total participants	Number of females
Kim et al. 2012 [30]	10,871,000	27.7%	4,862,670
Mathews et al. 2012 [31]	3,718,336	8.3%	1,463,276
Svedberg et al. 2019 [32]	736,102	1.5%	256,378
Arem et al. 2015 [33]	661,137	2.1%	369,652
Roberts et al. 2013 [34]	548,092	1.0%	168,227
Jin et al. 2019 [35]	501,690	1.4%	250,664
Hallmarker et al. 2016 [36]	204,038	0.4%	77,534
Belonje 2007 [37]	62,862	0.3%	47,775
Andersen et al. 2013 [9]	54,560	0.0%	5,443
Williams and Franklin 2013 [38]	46,907	0.2%	27,854

### Temporal Trends in Female Recruitment

**Fig. 2:**
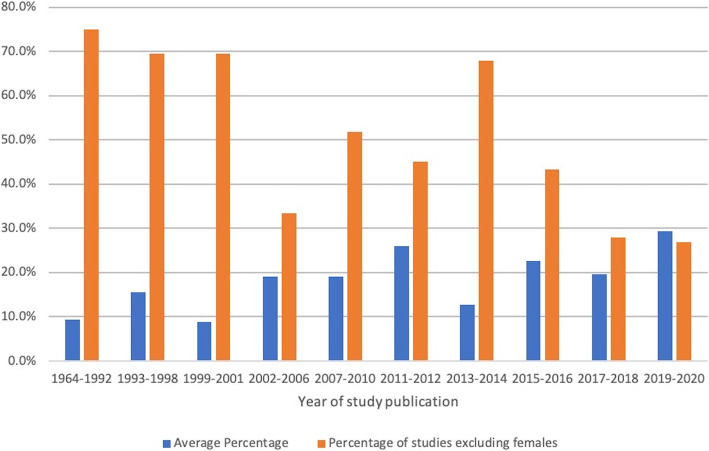
The mean percentage of female recruitment and percentage of studies excluding females over time periods to make equal study groups

The recruitment of females has changed over time. With the top ten largest studies excluded, 120 studies (56,256 participants) were completed before 2011 and 120 studies (87,863 participants) after (Table 1). Female participation demonstrated a positive trend, with a higher mean percentage and a lower number of studies excluding females after 2011. This trend was also seen when assessing recruitment characteristics of studies that have been categorised into ten time segments of approximately equal numbers of studies (Fig. 2). The 2019–2020 category had both the highest mean recruitment and the lowest percentage of female exclusion from all the groups.

### Sport, Regional and Outcome Trends

Running is the most commonly studied exercise type and makes up a large proportion of the studies in this literature base (43.3%, Table 3). This analysis provides some early evidence that female recruitment may vary between each exercise type. The highest recruitment (other category = 31.5%) had over twice the mean percentage of recruitment of the lowest (cross-country skiing = 12.6%) (Table 3). A similar variation is seen in the percentage of studies that exclude females altogether, with studies that assess running excluding 58.7% compared with 39.7% studies that assess mixed sports (Table 3).

**Table 3:** Recruitment of females by exercise type, WHO region and clinical outcome

	Number of studies	Number of studies excluding females	Percentage of studies excluding females (%)	Mean percentage females recruited per study (%)
Exercise type				
Running	109	64	58.7	14.5
Triathlon	25	12	48.0	14.0
Cross-country skiing	24	14	58.3	12.6
Mixed	63	25	39.7	22.3
Other	29	12	41.4	31.5
WHO region				
African region	2	2	100.0	0.0
Region of the Americas	67	26	38.8	27.6
Eastern Mediterranean region	0	0	NA	NA
European region	165	89	54.5	15.3
Southeast Asia region	0	0	NA	NA
Western Pacific region	16	10	62.5	11.5
Clinical outcome				
Arrhythmia	31	12	38.7	18.7
Cardiomyopathy	11	5	45.5	12.8
Ischaemic heart disease	10	8	80.0	6.2
Other	26	10	38.5	21.7

Most studies were conducted within the European Region and the Region of the Americas (Table 3). As a large proportion of studies occurred within these two regions, it is difficult to assess for descriptive trends in the other WHO regions. While not within the scope of this review, these results may serve as evidence that a large number of ethnicities and regions are not represented in this evidence base.

The proportion of studies excluding female participants was similar for all outcomes except ischaemic heart disease, where 80% of studies excluded females (Table 3). A similar trend was seen in mean percentage recruitment, where the lowest mean percentage (6.2%) was seen in studies that assessed ischaemic heart disease (Table 3).

## Discussion

The results from this study show that females are underrepresented in the literature that assesses the impact of high-dose exercise on cardiovascular outcomes. The striking finding here is that more than half the studies included in this review excluded females altogether. The numbers of female athletes participating at professional and nonprofessional levels are increasing [18, 39, 40], even among sports that are traditionally male dominated [41]. Large investments have been made into sports such as football and cycling, with the hope that this will improve access and participation [42, 43]. As participation grows, cardiovascular risk stratification in this population will need to be established such that risk stratifications are not extrapolated from largely male-recruited evidence. Equality in representation must become a priority.

There is no clear consensus on the need for cardiovascular risk stratification and screening services in athletes [44–46]. While there is a large body of evidence describing changes in potential cardiac markers, often termed as the athlete’s heart, the prognostic implications of this are less well understood [5]. Of all the hard-clinical endpoints, arrhythmias have shown the strongest evidence as a potential adverse clinical outcome of high-dose exercise. The results of this study confirm that arrhythmias were by far the most studied clinical outcome (39.0% of studies assessing clinical endpoints). A number of large observational studies have shown that athletes are at higher risk of AF [7, 47]. The management and risks of arrhythmias such as AF show variations between sex, with the female sex being a criterion used to determine anticoagulation requirement [48]. Management of AF in athletes is largely based on guidelines extrapolated from the general population, but it may be that the risk profiles and lifestyles of athletes warrant an alternative approach [47, 49]. The management of female athletes with AF is extrapolated further if, as shown in the results of this study, most of the literature focuses on males. While the risk of high-dose exercise for other clinical outcomes, such as ischaemic heart disease, is less clear, the same issues may persist if the scientific literature continues to underrecruit females. The underrecruitment of females in high-dose exercise may be seen in non-cardiovascular outcomes, too; this may be a future area for future studies.

The evidence for female populations presenting atypically with cardiovascular pathology is emerging [50]. If the findings of this review are applicable in other areas, then it may be that female representation is lacking in cardiovascular research altogether [51–53]. It could be that ‘atypical’ presentations are not atypical at all, and rather a reflection of the male-dominated recruitment seen in the literature. Evidence shows that cardiovascular risk is not the same between sexes. Therefore, policymakers should remain cautious when translating findings from male-dominated literature.

### Barriers to Female Recruitment

Barriers will need to be outlined if future studies are to improve recruitment of females in high-dose exercise cohorts. While this scoping review did not conduct statistical tests, the results from this study suggest that female recruitment may be improving (from 14.6% before 2011 to 21.6% after 2011). It is difficult to ascertain the direction of cause to explain this increase in recruitment; are more females specifically being recruited to reflect increased participation, or is it that recruitment of females is easier as participation levels increase? While recruitment of females has apparently increased, these are still far from the numbers seen in males. More than half the studies included in this review did not include females, but very few offered reasons for exclusion. For example, Myrstad et al. stated that the event which participants were recruited from had a low number of female participants; hence, they decided only to study male participants [54]. Some studies argue there to be limited differences between sexes in the outcome of interest [55]. It has also been reasoned that cardiac adaptations are more easily seen in male subjects [56]. While this may be true, it remains to be seen if these findings are translatable into female populations. It is also unclear if more easily detectable adaptations result in a poorer prognostic outcome.

Analysing temporal trends into ten time segments highlighted an increased focus on the impacts of high-dose exercise. As the groups were chosen to divide the studies into approximately equal sizes, it is interesting to see that the number of years that each group represents became smaller (1964–1992 versus 2019–2020). This may be a reflection of the recognition and priority that cardiovascular outcomes in high-dose exercise have received in recent years.

Exercise type, region and the outcome of interest may also influence levels of female recruitment seen. This review found a variation in the mean percentage of females recruited between sports (from 12.6% in cross-country skiing to 31.5% in “other”). Although not within the scope of this study, this may be a causal relationship, as sports with lower female participation may struggle to adequately recruit females. Sports with large, national funding bodies that have the administrative capacity to keep up-to-date athlete registries may favour female recruitment. For nonprofessionals, sports that have large events, such as marathons, allow investigators to recruit by the registration that athletes used to sign up. This is harder in types of exercise where large-scale, official events are less common. Population characteristics, related to the culture and socioeconomic profile of people, may also be a key barrier. In our analysis, 27.0% of studies were in the Region of the Americas and 65.9% in the European Region. Only 16 and 2 studies were conducted in the Western Pacific and African Regions respectively, while no studies were conducted in Southeast Asia or eastern Mediterranean regions. Lower and middle-income countries traditionally have lower levels of female participation, when compared with Western countries [57, 58]. Female participation in high-dose exercise may be less culturally favourable in some regions [59, 60]. These barriers to participation may translate into barriers to recruitment. This may be due to these groups being hard-to-reach, and scientific priorities may lie in investigating males as they are the larger participant group. However, as priorities and attitudes change, female participation may increase, highlighting the need for a well-represented evidence base [58]. Investigators should therefore consider specific, potential barriers to recruitment, such that the representation of females can be optimised. While not within the scope of this review, the lack of studies in Southeast Asia may be of concern to policymakers as cardiovascular risk is particularly high within this region [61].

Studies that assessed ischaemic heart disease demonstrated the poorest level of female recruitment. The reasons and direction of causality is unclear. In the wider literature, studies have reported disparities between sexes in ischaemic heart disease at multiple levels including diagnosis, reperfusion therapy, pharmacological therapy and prognosis [62]. Given this wider evidence, translating findings across sexes is not appropriate. As 80% of these studies excluded females altogether, the assessment of ischaemic heart disease and high-dose exercise may be a priority area for future research.

Solutions to sport, outcome and region-related barriers may come through the recruitment of individuals at large worldwide events such as the Olympics. It may be easier to recruit athletes from a range of sports and those from hard-to-reach groups at events like the Olympics. Studies evaluating nonprofessional participants may explore recruitment through other large-scale events [63]. A number of large marathon participation studies included in our analysis achieved fair levels of female representation adopting this method [30, 31, 64].

### Limitations

This review has limitations. Only peer reviewed literature was considered for the review. Findings presented through conferences are not considered in this analysis. This decision was made to ensure scientific quality in the evidence that this review would analyse. The coding of various sports is difficult, and so providing an exhaustive list of sports that may be included was not done. Instead, only studies that explicitly state that they looked at ‘high-dose’ exercise or studies that stated exercise type clearly associated with high dose (e.g. marathon running) were included. It may be that we missed studies that were not coded to be picked up by our search. A number of studies did not report the full data needed to fulfil our inclusion criteria. While this may introduce bias, we believe that this is likely to have led to overestimation of female recruitment. A number of studies that were excluded had elements, such as MeSH terms and descriptions, that suggested that they were male-exclusive, but as this was not explicitly stated, they were not included. As the study included both athletes and non-athlete individuals, this may introduce heterogeneity as the impacts of high-dose exercise on the cardiovascular system may vary between athletes and non-athletes. However, this review aims to assess how studies chose to recruit participants across the entire population. This review used a mean percentage of females per study for analysis rather than raw total data. While using mean percentage may give undue weighting to smaller studies, this is not a limitation within the scope of this review as it focuses on the recruitment of females, rather than any findings that each study reported. Taking a raw percentage may give undue weighting to these large studies that will mask true recruitment patterns. Large studies with a lower proportion of recruitment may have had enough absolute number of female participants to ensure that their analysis was powered to detect differences. This review did not collect other participant characteristics such as age. The extraction of these data may be a target for future reviews as this may reveal other participant-related barriers to female recruitment. Lastly, this review extracted both the high-dose group and controls from each study, with this decision allowing concentration on the recruitment of all individuals. It is likely that this strategy, again, led to an overestimation in the number of female athletes recruited as a number of studies reported very few females in the intervention arm, but more in the control arm [65, 66]. Furthermore, one study recruited females into the initial group, and so met the inclusion criteria for this review, but only chose to follow up on the male athletes [67]. Again, this suggests that this review may overestimate the recruitment of females.

## Conclusions

This scoping review sought to explore the representation of females in the literature assessing the impact of high-dose exercise on cardiovascular outcomes. It demonstrates that females are underrepresented when compared with males. This area of clinical research should undergo a paradigm shift to focus recruitment on a more representative sample of sexes. Future investigators should optimise recruitment strategy, possibly through the use of national sports body registries, in order to improve female recruitment into these studies.

## Supplementary information

**Additional file 1: Supplement 1** – PICO framework. **Supplement 2**: Search strategy. **Supplement 3** – Impact of excluding the largest studies. **Supplement 4** – Bibliography of all studies included in analysis. **Supplement 5** – Table showing characteristics of the studies included within the scoping review

## Data Availability

All data and materials were freely available.

## Abbreviations

PRISMA-ScR: Preferred reporting items for systematic reviews and meta-analyses extension for scoping reviews
FITT: Frequency, intensity, time and type
HR: Hazard ratio
AF: Atrial fibrillation
PICO: Population, intervention, controls, outcome
WHO: World Health Oraganization
IQR: Interquartile range
CAC: Coronary artery calcification
