# Supplementary material for: The Underrepresentation of Females in Studies Assessing the Impact of High-Dose Exercise on Cardiovascular Outcomes: a Scoping Review
Source: Sports Med Open. 2021 Apr 29;7:30. doi: 10.1186/s40798-021-00320-y (PMC8085142; doi:10.1186/s40798-021-00320-y)
Supplement: Supplementary file 1 — Additional file 1: Supplement 1 – PICO framework. Supplement 2: Search strategy. Supplement 3 – Impact of excluding the largest studies. Supplement 4 – Bibliography of all studies included in analysis. Supplement 5 – Table showing characteristics of the studies included within the scoping review [file 40798_2021_320_MOESM1_ESM.pdf]

Supplementary Documents

Supplement 1 – PICO framework

**Participants:**	**Intervention:**
All humans<br>Children<br>Adults<br>Males<br>Females<br>Athletes<br>Non-athletes	High dose/high intensity/high levels/ intensive/above recommended<br>Dose response?<br>Physical activity/exercise/training/
**Comparison:**<br>NA	**Outcomes:**<br>Arrhythmia<br>Cardiovascular outcomes<br>All-cause mortality<br>Ischaemic heart disease<br>Athletes heart<br>Cardiomyopathy<br>Cardiovascular disease

Supplement 2: Search strategy

OVID	EMBASE
1. (exercis* or physical activity or training or exercise training).mp.	1. (exercis* or physical activity or training or exercise training).ti,ab
2. ((high or prolong* or sustained or vigorous or increas*) and dos*).mp.	2. ((high or prolong* or sustained or vigorous or increas*) and dos*).ti,ab
3. (marathon or cross country ski* or iron man* or triath* or ultra distance or endurance cycl* or endurance row* or endurance kayak* or olympi* or professional athlete or elite performance).mp	3. (marathon or cross country ski* or iron man* or triath* or ultra distance or endurance cycl* or endurance row* or endurance kayak* or olympi* or professional athlete or elite performance).ti,ab
4. (colleg* or university or club or recreational).mp.	4. (colleg* or university or club or recreational).ti,ab
5. (cycl* or row* or swim* or kayak* or run* or aerobic or endurance).mp.	5. (cycl* or row* or swim* or kayak* or run* or aerobic or endurance).ti,ab
6. arrhythmia*.mp. or exp Arrhythmias, Cardiac/ or arrhythmia cardiac.mp.	6. arrhythmia*.ti,ab or exp Heart arrhythmia/ or arrhythmia cardiac.ti,ab
7. (tachyarrhythmia or tachycardia).mp. or exp Tachycardia/	7. (tachyarrhythmia or tachycardia).ti,ab or exp Tachycardia/
8. (bradycardia or bradyarrhythmia).mp. or exp Bradycardia/	8. (bradycardia or bradyarrhythmia).ti,ab or exp Bradycardia/
9. ECG chang*.mp.	9. ECG chang*.ti,ab
10. Heart block.mp. or exp Heart Block/	10. Heart block.ti,ab or exp Heart Block/
11. (cardiovascular disease* or heart disease*).mp. or Cardiovascular Diseases/ or cardiovascular outcome*.mp.	11. (cardiovascular disease* or heart disease*).ti,ab or Cardiovascular Disease/ or cardiovascular outcome*.ti,ab

12. (Cardiovascular adj5 mortality).mp.	12. (Cardiovascular adj5 mortality).ti,ab
13. isch* heart disease.mp. or Myocardial Ischaemia/ or myocardial isch*.mp. or Myocardial Infarction/	13. isch* heart disease.ti,ab or Heart muscle ischemia / or myocardial isch*.ti,ab or Heart infarction/
14. cardiomyopath*.mp. or exp Cardiomyopathies/	14. cardiomyopath*.ti,ab or exp Cardiomyopathy/
15. athlete* heart.mp. or Hypertrophy, Left Ventricular/ or Hypertrophy, Right Ventricular/ or Arrhythmogenic Right Ventricular Dysplasia/ or Cardiomyopathy, Hypertrophic/ or Cardiomyopathy, Restrictive/ or Cardiomyopathy, Dilated/	15. athlete* heart.ti,ab or exp Heart hypertrophy/ or heart right ventricle dysplasia/
16. heart failure.mp. or Heart Failure/	16. heart failure.ti,ab or exp Heart Failure/
17. 1 and 2	17. 1 and 2
18. 4 and 17	18. 4 and 17
19. 5 and 17	19. 5 and 17
20. 6 or 7 or 8 or 9 or 10 or 11 or 12 or 13 or 14 or 15 or 16	20. 6 or 7 or 8 or 9 or 10 or 11 or 12 or 13 or 14 or 15 or 16
21. 3 or 18 or 19	21. 3 or 18 or 19
22. 20 and 21	22. 20 and 21

Supplement 3 – Impact of excluding the largest studies

Number of Studies Excluded (in size order)	Percentage Female Recruitment
1	40.3%
2	41.6%
3	43.8%
4	38.7%
5	43.0%
6	36.2%
7	35.1%
8	24.6%
9	28.8%
10	18.8%
11	14.8%
12	16.4%
13	17.9%
14	19.6%
15	21.5%
16	19.0%
17	20.6%
18	21.6%
19	20.6%
20	22.1%

Supplement 4 – Bibliography of all studies included in analysis

1. Abdullah SM, Barkley KW, Bhella PS, et al. Lifelong Physical Activity Regardless of Dose Is Not Associated With Myocardial Fibrosis. Circulation Cardiovascular imaging 2016;9(11)

2. Aengevaeren VL, Mosterd A, Braber TL, et al. Relationship Between Lifelong Exercise Volume and Coronary Atherosclerosis in Athletes. Circulation 2017;136(2):138-48.

3. Andersen K, Farahmand B, Ahlbom A, et al. Risk of arrhythmias in 52 755 long-distance cross-country skiers: a cohort study. European heart journal 2013;34(47):3624-31.

4. Andersson J, Jansson J-H, Hellsten G, et al. Effects of heavy endurance physical exercise on inflammatory markers in non-athletes. Atherosclerosis 2010;209(2):601-5.

5. Antero-Jacquemin J, Desgorces FD, Dor F, et al. Row for your life: a century of mortality follow-up of French olympic rowers. PloS one 2014;9(11):e113362.

6. Antero-Jacquemin J, Pohar-Perme M, Rey G, et al. The heart of the matter: years-saved from cardiovascular and cancer deaths in an elite athlete cohort with over a century of follow-up. European journal of epidemiology 2018;33(6):531-43.

7. Antero-Jacquemin J, Rey G, Marc A, et al. Mortality in female and male French Olympians: a 1948-2013 cohort study. The American journal of sports medicine 2015;43(6):1505-12.

8. Apple FS, McGue MK. Serum enzyme changes during marathon training. American Journal of Clinical Pathology 1983;79(6):716-9.

9. Apple FS, Quist HE, Otto AP, et al. Release characteristics of cardiac biomarkers and ischemia-modified albumin as measured by the albumin cobalt-binding test after a marathon race. Clinical chemistry 2002;48(7):1097-100.

10. Apple FS, Rogers MA, Sherman WM, et al. Comparison of serum creatine kinase and creatine kinase MB activities post marathon race versus post myocardial infarction. Clinica chimica acta; international journal of clinical chemistry 1984;138(1):111-8.

11. Arem H, Moore SC, Patel A, et al. Leisure time physical activity and mortality: a detailed pooled analysis of the dose-response relationship. JAMA internal medicine 2015;175(6):959-67.

12. Banfi G, Lippi G, Susta D, et al. NT-proBNP concentrations in mountain marathoners. Journal of strength and conditioning research 2010;24(5):1369-72.

13. Banfi G, Migliorini S, Dolci A, et al. B-type natriuretic peptide in athletes performing an Olympic triathlon. The Journal of sports medicine and physical fitness 2005;45(4):529-31.

14. Belonje A, Nangrahary M, de Swart H, et al. Major adverse cardiac events during endurance sports. The American journal of cardiology 2007;99(6):849-51.

15. Bennekers JH, van Mechelen R, Meijer A. Pacemaker safety and long-distance running. Netherlands heart journal : monthly journal of the Netherlands Society of Cardiology and the Netherlands Heart Foundation 2004;12(10):450-54.

16. Bergstrom E, Hernell O, Persson LA. Endurance running performance in relation to cardiovascular risk indicators in adolescents. International journal of sports medicine 1997;18(4):300-7.

17. Bernardino G, Sanz de la Garza M, Domenech-Ximenos B, et al. Three-dimensional regional bi-ventricular shape remodeling is associated with exercise capacity in endurance athletes. European journal of applied physiology 2020;120(6):1227-35.

18. Biffi A, Ansalone G, Verdile L, et al. Ventricular arrhythmias and athlete's heart. Role of signal-averaged electrocardiography. Eur Heart J 1996;17(4):557-63. doi: 10.1093/oxfordjournals.eurheartj.a014909

19. Biffi A, Verdile L, Ansalone G, et al. Lack of correlation between ventricular late potentials and left ventricular mass in top-level male athletes. Med Sci Sports Exerc 1999;31(3):359-61. doi: 10.1097/00005768-199903000-00001

20. Bjerring AW, Landgraff HE, Leirstein S, et al. Morphological changes and myocardial function assessed by traditional and novel echocardiographic methods in preadolescent athlete's heart. European journal of preventive cardiology 2018;25(9):1000-07.

21. Bjerring AW, Landgraff HE, Leirstein S, et al. From talented child to elite athlete: The development of cardiac morphology and function in a cohort of endurance athletes from age 12 to 18. European journal of preventive cardiology 2020:2047487320921317.

22. Bjerring AW, Landgraff HE, Stokke TM, et al. The developing athlete's heart: a cohort study in young athletes transitioning through adolescence. European journal of preventive cardiology 2019;26(18):2001-08.

23. Bonsignore A, Bredin SSD, Wollmann H, et al. The influence of race length on arterial compliance following an ultra-endurance marathon. European journal of sport science 2017;17(4):441-46.

24. Breuckmann F, Mohlenkamp S, Sievers B, et al. Myocardial late gadolinium enhancement: Prevalence, pattern, and prognostic relevance in marathon runners. 2009;251(1):50-57.

25. Calvo N, Ramos P, Montserrat S, et al. Emerging risk factors and the dose-response relationship between physical activity and lone atrial fibrillation: a prospective case-control study. Europace : European pacing, arrhythmias, and cardiac electrophysiology : journal of the working groups on cardiac pacing, arrhythmias, and cardiac cellular electrophysiology of the European Society of Cardiology 2016;18(1):57-63.

26. Carlsson M, Ugander M, Mosen H, et al. Atrioventricular plane displacement is the major contributor to left ventricular pumping in healthy adults, athletes, and patients with dilated cardiomyopathy. American journal of physiology Heart and circulatory physiology 2007;292(3):H1452-9.

27. Caselli S, Autore C, Serdoz A, et al. Three-dimensional echocardiographic characterization of patients with left ventricular noncompaction. Journal of the American Society of Echocardiography : official publication of the American Society of Echocardiography 2012;25(2):203-9.

28. Caselli S, Di Paolo FM, Pisicchio C, et al. Patterns of left ventricular diastolic function in Olympic athletes. Journal of the American Society of Echocardiography : official publication of the American Society of Echocardiography 2015;28(2):236-44.

29. Caselli S, Di Paolo FM, Pisicchio C, et al. Patterns of left ventricular longitudinal strain and strain rate in olympic athletes. 2015;28(2):245-53.

30. Claessens C, Claessens P, Bloemen H, et al. Structural heart adaptations in triathletes. Acta cardiologica 1999;54(6):317-25.

31. Claessens C, Claessens P, Claessens M, et al. Echocardiographic and physiological performance characteristics of triathletes. 2000;16(8):993-1002.

32. Claessens P, Claessens C, Claessens M, et al. Ventricular prematuue beats in triathletes: Still a physiological phenomenon? 1999;92(1):28-38.

33. Claessens P, Claessens C, Claessens M, et al. Physiological or pseudophysiological ECG changes in endurance-trained athletes. Heart and vessels 2000;15(4):181-90.

34. Claessens PJM, Claessens CWF, Claessens MMM, et al. Supernormal left ventricular diastolic function in triathletes. 2001;28(2):102-10.

35. Clauss S, Wakili R, Hildebrand B, et al. MicroRNAs as Biomarkers for Acute Atrial Remodeling in Marathon Runners (The miRathon Study--A Sub-Study of the Munich Marathon Study). PloS one 2016;11(2):e0148599.

36. Conti V, Corbi G, Russomanno G, et al. Oxidative stress effects on endothelial cells treated with different athletes' sera. Medicine and science in sports and exercise 2012;44(1):39-49.

37. Cubeddu LX, Rodriguez-Plaza LG, Alfieri AB. Urinary excretion of nitric oxide metabolites in runners, sedentary individuals and patients with coronary artery disease: Effects of 42 km marathon, 15 km race and a cardiac rehabilitation program. 1997;4(5):367-72.

38. D'Ascenzi F, Caselli S, Alvino F, et al. Cardiovascular risk profile in Olympic athletes: an unexpected and underestimated risk scenario. British journal of sports medicine 2019;53(1):37-42.

39. D'Ascenzi F, Pisicchio C, Caselli S, et al. RV Remodeling in Olympic Athletes. JACC Cardiovascular imaging 2017;10(4):385-93.

40. D'Silva A, Bhuva AN, van Zalen J, et al. Cardiovascular Remodeling Experienced by Real-World, Unsupervised, Young Novice Marathon Runners. Frontiers in physiology 2020;11:232.

41. D'Silva A, Captur G, Bhuva AN, et al. Recreational marathon running does not cause exercise-induced left ventricular hypertrabeculation. International journal of cardiology 2020

42. Dalla Vecchia L, Traversi E, Porta A, et al. On site assessment of cardiac function and neural regulation in amateur half marathon runners. 2014;1(1)

43. Damm S, Andersson LG, Henriksen E, et al. Wall motion abnormalities in male elite orienteers are aggravated by exercise. Clinical physiology (Oxford, England) 1999;19(2):121-6.

44. Date H, Imamura T, Onitsuka H, et al. Differential increase in natriuretic peptides in elite dynamic and static athletes. Circulation journal : official journal of the Japanese Circulation Society 2003;67(8):691-6.

45. Davis CK, Dyar DA, Vargas LA, et al. Cardiovascular and Musculoskeletal Assessment of Elite US Volleyball Players. Clinical journal of sport medicine : official journal of the Canadian Academy of Sport Medicine 2015;25(6):546-50.

46. Dawkins TG, Shave RE, Baggish AL, et al. Electrocardiographic changes following six months of long-distance triathlon training in previously recreationally active individuals. European journal of sport science 2019:1-10.

47. Demirelli S, Sam CT, Ermis E, et al. Long-Term Cardiac Remodeling in Elite Athletes: Assessment by Tissue Doppler and Speckle Tracking Echocardiography. Echocardiography (Mount Kisco, NY) 2015;32(9):1367-73.

48. Denham J, Nelson CP, O'Brien BJ, et al. Longer leukocyte telomeres are associated with ultra-endurance exercise independent of cardiovascular risk factors. PloS one 2013;8(7):e69377.

49. Deshaies Y, Allard C. Serum high-density lipoprotein cholesterol in male and female Olympic athletes. Medicine and science in sports and exercise 1982;14(3):207-11.

50. Draheim CC, Williams DP, McCubbin JA. Cardiovascular disease risk factor differences between Special Olympians and Non-Special Olympians. 2003;20(2):118-33.

51. Dressendorfer RH, Hauser AM, Timmis GC. Reversal of runner's bradycardia with training overstress. Clinical journal of sport medicine : official journal of the Canadian Academy of Sport Medicine 2000;10(4):279-85.

52. Dupont A-C, Poussel M, Hossu G, et al. Aortic compliance variation in long male distance triathletes: A new insight into the athlete's artery? Journal of science and medicine in sport 2017;20(6):539-42.

53. Durand G, Tsismenakis AJ, Jahnke SA, et al. Firefighters' physical activity: relation to fitness and cardiovascular disease risk. Medicine and science in sports and exercise 2011;43(9):1752-9.

54. Edwards M, Loprinzi P. The Dose-Response Association Between Reported Moderate to Vigorous Intensity Physical Activity and Atherogenic Index of Plasma: NHANES, 1999-2006. Journal of physical activity & health 2019;16(5):368-70.

55. Franco V, Callaway C, Salcido D, et al. Characterization of electrocardiogram changes throughout a marathon. European journal of applied physiology 2014;114(8):1725-35.

56. Franzen E, Mangold S, Erz G, et al. Comparison of morphological and functional adaptations of the heart in highly trained triathletes and long-distance runners using cardiac magnetic resonance imaging. Heart and vessels 2013;28(5):626-31.

57. Furlanello F, Bertoldi A, Dallago M, et al. Atrial fibrillation in elite athletes. Journal of cardiovascular electrophysiology 1998;9(8 Suppl):S63-8.

58. Gabrielli L, Herrera S, Contreras-Briceno F, et al. Increased active phase atrial contraction is related to marathon runner performance. European journal of applied physiology 2018;118(9):1931-39.

59. Galetta F, Franzoni F, Santoro G, et al. QT dispersion in elderly athletes with left ventricular hypertrophy. Int J Sports Med 2003;24(4):233-7. doi: 10.1055/s-2003-39499

60. Gaudreault V, Tizon-Marcos H, Poirier P, et al. Transient myocardial tissue and function changes during a marathon in less fit marathon runners. The Canadian journal of cardiology 2013;29(10):1269-76.

61. Ginsburg GS, Agil A, O'Toole M, et al. Effects of a single bout of ultraendurance exercise on lipid levels and susceptibility of lipids to peroxidation in triathletes. JAMA 1996;276(3):221-5.

62. Ginsburg GS, O'Toole M, Rimm E, et al. Gender differences in exercise-induced changes in sex hormone levels and lipid peroxidation in athletes participating in the Hawaii Ironman triathlon. Ginsburg-gender and exercise-induced lipid peroxidation. Clinica chimica acta; international journal of clinical chemistry 2001;305(1-2):131-9.

63. Grabs V, Peres T, Zelger O, et al. Decreased prevalence of cardiac arrhythmias during and after vigorous and prolonged exercise in healthy male marathon runners. American heart journal 2015;170(1):149-55.

64. Gram AS, Bladbjerg E-M, Skov J, et al. Three months of strictly controlled daily endurance exercise reduces thrombin generation and fibrinolytic risk markers in younger moderately overweight men. European journal of applied physiology 2015;115(6):1331-8.

65. Grimsmo J, Grundvold I, Maehlum S, et al. High prevalence of atrial fibrillation in long-term endurance cross-country skiers: echocardiographic findings and possible predictors--a 28-30 years follow-up study. European journal of cardiovascular prevention and rehabilitation : official journal of the European Society of Cardiology, Working Groups on Epidemiology & Prevention and Cardiac Rehabilitation and Exercise Physiology 2010;17(1):100-5.

66. Grimsmo J, Maehlum S, Moelstad P, et al. Mortality and cardiovascular morbidity among long-term endurance male cross country skiers followed for 28-30 years. Scandinavian journal of medicine & science in sports 2011;21(6):e351-8.

67. Haeusler KG, Herm J, Kunze C, et al. Rate of cardiac arrhythmias and silent brain lesions in experienced marathon runners: rationale, design and baseline data of the Berlin Beat of Running study. BMC cardiovascular disorders 2012;12:69.

68. Hallmarker U, Michaelsson K, Arnlov J, et al. Risk of recurrent ischaemic events after myocardial infarction in long-distance ski race participants. European journal of preventive cardiology 2016;23(3):282-90.

69. Hanssen H, Keithahn A, Hertel G, et al. Magnetic resonance imaging of myocardial injury and ventricular torsion after marathon running. Clinical science (London, England : 1979) 2011;120(4):143-52.

70. Harris KM, Creswell LL, Haas TS, et al. Death and Cardiac Arrest in U.S. Triathlon Participants, 1985 to 2016: A Case Series. Annals of internal medicine 2017;167(8):529-35.

71. Hattasch R, Spethmann S, de Boer RA, et al. Galectin-3 increase in endurance athletes. European journal of preventive cardiology 2014;21(10):1192-9.

72. Hawkins MN, Barnes Q, Purkayastha S, et al. The effects of aerobic fitness and beta1-adrenergic receptor blockade on cardiac work during dynamic exercise. Journal of applied physiology (Bethesda, Md : 1985) 2009;106(2):486-93.

73. Heinz L, Sax A, Robert F, et al. T-wave variability detects abnormalities in ventricular repolarization: a prospective study comparing healthy persons and Olympic athletes. Annals of noninvasive electrocardiology : the official journal of the International Society for Holter and Noninvasive Electrocardiology, Inc 2009;14(3):276-9.

74. Henriksen E, Landelius J, Wesslen L, et al. Echocardiographic right and left ventricular measurements in male elite endurance athletes. European heart journal 1996;17(7):1121-8.

75. Her AY, Kim J-Y, Choi E-Y, et al. Value of ventricular stiffness index and ventriculoarterial interaction in patients with nonischemic dilated cardiomyopathy. Circulation journal : official journal of the Japanese Circulation Society 2009;73(9):1683-90.

76. Herm J, Topper A, Wutzler A, et al. Frequency of exercise-induced ST-T-segment deviations and cardiac arrhythmias in recreational endurance athletes during a marathon race: results of the prospective observational Berlin Beat of Running study. BMJ open 2017;7(8):e015798.

77. Herrmann M, Schorr H, Obeid R, et al. Homocysteine increases during endurance exercise. 2003;41(11):1518-24.

78. Hildick-Smith DJ, Johnson PJ, Wisbey CR, et al. Coronary flow reserve is supranormal in endurance athletes: an adenosine transthoracic echocardiographic study. Heart 2000;84(4):383-9. doi: 10.1136/heart.84.4.383

79. Hirata Y, Kusunose K, Yamada H, et al. Left atrial functional response after a marathon in healthy amateur volunteers. The international journal of cardiovascular imaging 2019;35(4):633-43.

80. Hochsmann C, Dorling JL, Apolzan JW, et al. Effect of different doses of supervised aerobic exercise on heart rate recovery in inactive adults who are overweight or obese: results from E-MECHANIC. European journal of applied physiology 2019;119(9):2095-103.

81. Hosatte-Ducassy C, Mohindra R, Marton G, et al. Electrocardiographic changes after completion of a triathlon. 2020

82. Iellamo F, Manzi V, Caminiti G, et al. Dose-response relationship of baroreflex sensitivity and heart rate variability to individually-tailored exercise training in patients with heart failure. International journal of cardiology 2013;166(2):334-9.

83. Iellamo F, Manzi V, Caminiti G, et al. Matched dose interval and continuous exercise training induce similar cardiorespiratory and metabolic adaptations in patients with heart failure. International journal of cardiology 2013;167(6):2561-5.

84. Imai K, Sato H, Hori M, et al. Vagally mediated heart rate recovery after exercise is accelerated in athletes but blunted in patients with chronic heart failure. Journal of the American College of Cardiology 1994;24(6):1529-35.

85. Indermuhle A, Vogel R, Meier P, et al. The relative myocardial blood volume differentiates between hypertensive heart disease and athlete's heart in humans. European heart journal 2006;27(13):1571-8.

86. Indermuhle A, Vogel R, Rutz T, et al. Myocardial contrast echocardiography for the distinction of hypertrophic cardiomyopathy from athlete's heart and hypertensive heart disease. Swiss medical weekly 2009;139(47-48):691-8.

87. Iwasaki K-I, Zhang R, Zuckerman JH, et al. Dose-response relationship of the cardiovascular adaptation to endurance training in healthy adults: how much training for what benefit? Journal of applied physiology (Bethesda, Md : 1985) 2003;95(4):1575-83.

88. Jin M-N, Yang P-S, Song C, et al. Physical Activity and Risk of Atrial Fibrillation: A Nationwide Cohort Study in General Population. Scientific reports 2019;9(1):13270.

89. Jouffroy R, Caille V, Vieillard-Baron A, et al. Changes of cardiac function during ultradistance trail running. 2015;116(8):1284-89.

90. Kaleta AM, Lewicka E, Dabrowska-Kugacka A, et al. Electrocardiographic abnormalities in amateur male marathon runners. Advances in clinical and experimental medicine : official organ Wroclaw Medical University 2018;27(8):1091-98.

91. Karlstedt E, Chelvanathan A, Da Silva M, et al. The impact of repeated marathon running on cardiovascular function in the aging population. Journal of cardiovascular magnetic resonance : official journal of the Society for Cardiovascular Magnetic Resonance 2012;14:58.

92. Karstoft K, Solomon TP, Laye MJ, et al. Daily marathon running for a week--the biochemical and body compositional effects of participation. Journal of strength and conditioning research 2013;27(11):2927-33.

93. Kasikcioglu E, Oflaz H, Oncul A, et al. The trivest in overreaching syndrome: cardiac fatigue, muscular weakness, and vascular dysfunction. International journal of cardiology 2008;127(3):417-9.

94. Ketelhut RG, Ketelhut K, Messerli FH, et al. Fitness in the fit: does physical conditioning affect cardiovascular risk factors in middle-aged marathon runners? European heart journal 1996;17(2):199-203.

95. Kettunen JA, Kujala UM, Kaprio J, et al. All-cause and disease-specific mortality among male, former elite athletes: an average 50-year follow-up. British journal of sports medicine 2015;49(13):893-7.

96. Kim JH, Malhotra R, Chiampas G, et al. Cardiac arrest during long-distance running races. The New England journal of medicine 2012;366(2):130-40.

97. Kim YJ, Goh CW, Byun YS, et al. Left ventricular hypertrophy, diastolic dysfunction, pulse pressure, and plasma ET-1 in marathon runners with exaggerated blood pressure response. International heart journal 2013;54(2):82-7.

98. Kim YJ, Park Y, Lee YH, et al. Relationship of exercise habits and cardiac events to resting blood pressure in middle-aged amateur marathoners. 2014;173(5):273-81.

99. Kokkinos PF, Holland JC, Narayan P, et al. Miles run per week and HDL-C levels in healthy, middle-aged men: A dose- response relationship. 1995;16(8):10-15.

100. Koller A, Sumann G, Griesmacher A, et al. Cardiac troponins after a downhill marathon. 2008;129(3):449-52.

101. Konopka M, Burkhard-Jagodzinska K, Aniol-Strzyzewska K, et al. Prevalence and determinants of the early repolarisation pattern in a group of young high endurance rowers. Kardiologia polska 2016;74(3):289-99.

102. Kostka T, Drygas W, Mrozek P. [Physiological characteristics and the level of risk factors for ischemic heart disease in marathon runners between 30 and 59 years of age]. Charakterystyka fizjologiczna oraz poziom czynnikow zagrozenia choroba niedokrwienna serca u biegaczy maratonczykow w wieku 30-59 lat 1989;44(6):144-6.

103. Krol W, Braksator W, Kasprzak JD, et al. The influence of extreme mixed exertion load on the right ventricular dimensions and function in elite athletes: a tissue Doppler study. Echocardiography (Mount Kisco, NY) 2011;28(7):753-60.

104. Krumm P, Kraus S, Mangold S, et al. Mid-term development of the right ventricle in competitive athletes. Acta radiologica (Stockholm, Sweden : 1987) 2018;59(12):1422-30.

105. Kunutsor SK, Makikallio TH, Kauhanen J, et al. Leisure-time cross-country skiing is associated with lower incidence of type 2 diabetes: A prospective cohort study. Diabetes/metabolism research and reviews 2020;36(1):e3216.

106. Kunutsor SK, Makikallio TH, Kauhanen J, et al. Leisure-time cross-country skiing is associated with lower incidence of hypertension: a prospective cohort study. Journal of hypertension 2019;37(8):1624-32.

107. Lacey B, Golledge J, Yeap BB, et al. Physical activity and vascular disease in a prospective cohort study of older men: The Health In Men Study (HIMS). BMC geriatrics 2015;15:164.

108. Lalande S, Baldi JC. Left ventricular mass in elite olympic weight lifters. The American journal of cardiology 2007;100(7):1177-80.

109. Leckie T, Richardson A, Watkins E, et al. High-sensitivity troponin T in marathon runners, marathon runners with heart disease and collapsed marathon runners. Scandinavian journal of medicine & science in sports 2019;29(5):663-68.

110. Legaz Arrese A, Serrano Ostariz E, Gonzalez Carretero M, et al. Echocardiography to measure fitness of elite runners. Journal of the American Society of Echocardiography : official publication of the American Society of Echocardiography 2005;18(5):419-26.

111. Leischik R, Spelsberg N. Endurance sport and "cardiac injury": A prospective study of recreational ironman athletes. 2014;11(9):9082-100.

112. Leischik R, Spelsberg N, Niggemann H, et al. Exercise-induced arterial hypertension - an independent factor for hypertrophy and a ticking clock for cardiac fatigue or atrial fibrillation in athletes? 2014;3

113. Lemez S, Wattie N, Baker J. Early death in active professional athletes: Trends and causes. Scandinavian journal of medicine & science in sports 2016;26(5):544-9.

114. Lewicka-Potocka Z, Dabrowska-Kugacka A, Lewicka E, et al. The "athlete's heart" features in amateur male marathon runners. Cardiology journal 2020

115. Lie H, Erikssen J. ECG aberrations, latent coronary heart disease and cardiopulmonary fitness in various age groups of Norwegian cross-country skiers. Acta medica Scandinavica 1978;203(6):503-7.

116. Lie H, Erikssen J. Five-year follow-up of ECG aberrations, latent coronary heart disease and cardiopulmonary fitness in various age groups of Norwegian cross-country skiers. Acta medica Scandinavica 1984;216(4):377-83.

117. Lie H, Mundal R, Erikssen J. Coronary risk factors and incidence of coronary death in relation to physical fitness. Seven-year follow-up study of middle-aged and elderly men. European heart journal 1985;6(2):147-57.

118. Lippi G, Schena F, Salvagno GL, et al. Comparison of the lipid profile and lipoprotein(a) between sedentary and highly trained subjects. Clinical chemistry and laboratory medicine 2006;44(3):322-6.

119. Lippi G, Schena F, Salvagno GL, et al. Influence of a half-marathon run on NT-proBNP and troponin T. Clinical laboratory 2008;54(7-8):251-4.

120. Lippi G, Schena F, Salvagno GL, et al. A half-marathon run does not acutely influence lipoprotein(a). Nutrition, metabolism, and cardiovascular diseases : NMCD 2008;18(7):e37-8.

121. Loprinzi PD. Dose-response association of moderate-to-vigorous physical activity with cardiovascular biomarkers and all-cause mortality: Considerations by individual sports, exercise and recreational physical activities. Preventive medicine 2015;81:73-7.

122. Lord R, Somauroo J, Stembridge M, et al. The right ventricle following ultra-endurance exercise: insights from novel echocardiography and 12-lead electrocardiography. European journal of applied physiology 2015;115(1):71-80.

123. Lucia A, Moran M, Perez M, et al. Short-term effects of marathon running in master runners: no evidence of myocardial injury. International journal of sports medicine 1999;20(7):482-6.

124. Luurila OJ, Karjalainen J, Viitasalo M, et al. Arrhythmias and ST segment deviation during prolonged exhaustive exercise (ski marathon) in healthy middle-aged men. European heart journal 1994;15(4):507-13.

125. Major Z, Kirschner R, Medvegy N, et al. The importance of ST elevation in V2-4 ECG leads in athletes. Physiology international 2019;106(4):368-78.

126. Makan J, Sharma S, Firoozi S, et al. Physiological upper limits of ventricular cavity size in highly trained adolescent athletes. Heart (British Cardiac Society) 2005;91(4):495-9.

127. Malek LA, Barczuk-Falecka M, Werys K, et al. Cardiovascular magnetic resonance with parametric mapping in long-term ultra-marathon runners. European journal of radiology 2019;117:89-94.

128. Mango F, Caselli S, Luchetti A, et al. Low QRS voltages in Olympic athletes: Prevalence and clinical correlates. European journal of preventive cardiology 2020:2047487320914758.

129. Marijon E, Tafflet M, Antero-Jacquemin J, et al. Mortality of French participants in the Tour de France (1947-2012). European heart journal 2013;34(40):3145-50.

130. Maron BJ, Klues HG. Surviving competitive athletics with hypertrophic cardiomyopathy. The American journal of cardiology 1994;73(15):1098-104.

131. Maron BJ, Pelliccia A, Spataro A, et al. Reduction in left ventricular wall thickness after deconditioning in highly trained Olympic athletes. British heart journal 1993;69(2):125-8.

132. Martin-Beaulieu JP, Myrick KM, Martin T, et al. Common electrocardiogram variations pre- and post-marathon. Clinical case reports 2016;4(10):944-47.

133. Martinez-Mas ML, Javier Chorro F, Sanchis J, et al. Doppler assessment of left ventricular diastolic function in marathon runners. The Journal of sports medicine and physical fitness 1994;34(1):50-5.

134. Mathews SC, Narotsky DL, Bernholt DL, et al. Mortality among marathon runners in the United States, 2000-2009. The American journal of sports medicine 2012;40(7):1495-500.

135. Matsuo S, Nakamura Y, Takahashi M, et al. Cardiac sympathetic dysfunction in an athlete's heart detected by 123I-metaiodobenzylguanidine scintigraphy. Japanese circulation journal 2001;65(5):371-4.

136. Mattusch F, Dufaux B, Heine O, et al. Reduction of the plasma concentration of C-reactive protein following nine months of endurance training. International journal of sports medicine 2000;21(1):21-4.

137. Mayet J, Kanagaratnam P, Shahi M, et al. QT dispersion in athletic left ventricular hypertrophy. American heart journal 1999;137(4 Pt 1):678-81.

138. McClean G, George K, Lord R, et al. Chronic adaptation of atrial structure and function in elite male athletes. European heart journal cardiovascular Imaging 2015;16(4):417-22.

139. Merghani A, Cox AT, Dhutia H, et al. Prevalence of Subclinical Coronary Artery Disease in Masters Endurance Athletes with a Low Atherosclerotic Risk Profile. 2017;136(2):126-37.

140. Middleton N, Shave R, George K, et al. Novel application of flow propagation velocity and ischaemia-modified albumin in analysis of postexercise cardiac function in man. Experimental physiology 2006;91(3):511-9.

141. Milvy P. Statistical analysis of deaths from coronary heart disease anticipated in a cohort of marathon runners. Annals of the New York Academy of Sciences 1977;301:620-6.

142. Minns AB, McFarland C, Strachan M, et al. Electrocardiogram and echocardiogram findings in runners completing a half marathon. The American journal of emergency medicine 2011;29(9):1182-7.

143. Miranda JO, Lopez DT. Modificaciones anatomicas del ventriculo izquierdo en deportistas de canotaje de alto rendimientoAnatomical modifications of the left ventricle in high performance canoeing athletes. 2020;19(1):76-91.

144. Mohlenkamp S, Leineweber K, Lehmann N, et al. Coronary atherosclerosis burden, but not transient troponin elevation, predicts long-term outcome in recreational marathon runners. Basic research in cardiology 2014;109(1):391.

145. Molina L, Mont L, Marrugat J, et al. Long-term endurance sport practice increases the incidence of lone atrial fibrillation in men: a follow-up study. Europace : European pacing, arrhythmias, and cardiac electrophysiology : journal of the working groups on cardiac pacing, arrhythmias, and cardiac cellular electrophysiology of the European Society of Cardiology 2008;10(5):618-23.

146. Montiel G, Horn T, Vafa R, et al. Echocardiographic and biochemical analysis of cardiac function and injury among female amateur runners post-marathon. 2016;128(5):193-97.

147. Morris JN, Clayton DG, Everitt MG, et al. Exercise in leisure time: coronary attack and death rates. British heart journal 1990;63(6):325-34.

148. Murayama M, Kuroda Y. Cardiovascular future of athletes. 1980;29(2):117-23.

149. Musat CL, Coman M, Hincu M, et al. Etude sur les effets du stress cause par l'effort aerobe sur la fonction cardiovasculaire et la fonction hepatique des athletes specialisees en probes de fond et grand fondA study on the effects of stress due to aerobic exertion on the cardiovascular and liver functions of athletes specializing in track running and long distance running. 2007;42(4):234-38.

150. Myrstad M, Aaronaes M, Graff-Iversen S, et al. Physical activity, symptoms, medication and subjective health among veteran endurance athletes with atrial fibrillation. Clinical research in cardiology : official journal of the German Cardiac Society 2016;105(2):154-61.

151. Myrstad M, Lochen ML, Graff-Iversen S, et al. Increased risk of atrial fibrillation among elderly Norwegian men with a history of long-term endurance sport practice. Scandinavian journal of medicine & science in sports 2014;24(4):e238-44.

152. Myrstad M, Nystad W, Graff-Iversen S, et al. Effect of years of endurance exercise on risk of atrial fibrillation and atrial flutter. The American journal of cardiology 2014;114(8):1229-33.

153. Nakamoto K. Electrocardiograms of 25 marathon runners before and after 100 meter dash. Japanese circulation journal 1969;33(2):105-28.

154. Nassenstein K, Breuckmann F, Lehmann N, et al. Left ventricular volumes and mass in marathon runners and their association with cardiovascular risk factors. The international journal of cardiovascular imaging 2009;25(1):71-9.

155. Nottin S, Doucende G, Tanguy S, et al. Alteration in left ventricular strains and torsional mechanics after ultralong duration exercise in athletes. 2009;2(4):323-30.

156. O'Hanlon R, Wage R, Smith G, et al. Troponin release following endurance exercise: Is inflammation the cause? A cardiovascular magnetic resonance study. 2010;12(1)

157. Ogawa S, Tabata H, Ohishi S, et al. Prognostic significance of long ventricular pauses in athletes. Japanese circulation journal 1991;55(8):761-6.

158. Ohba H, Takada H, Musha H, et al. Effects of prolonged strenuous exercise on plasma levels of atrial natriuretic peptide and brain natriuretic peptide in healthy men. American heart journal 2001;141(5):751-8.

159. Ohman EM, Teo KK, Johnson AH, et al. Abnormal cardiac enzyme responses after strenuous exercise: alternative diagnostic aids. British medical journal (Clinical research ed) 1982;285(6354):1523-6.

160. Olivier LR, de Waal A, Retief FJ, et al. Electrocardiographic and biochemical studies on marathon runners. South African medical journal = Suid-Afrikaanse tydskrif vir geneeskunde 1978;53(20):783-7.

161. Opondo MA, Aiad N, Cain MA, et al. Does High-Intensity Endurance Training Increase the Risk of Atrial Fibrillation? A Longitudinal Study of Left Atrial Structure and Function. Circulation Arrhythmia and electrophysiology 2018;11(5):e005598.

162. Paffenbarger RS, Jr., Hyde RT, Wing AL, et al. A natural history of athleticism and cardiovascular health. JAMA 1984;252(4):491-5.

163. Palazzuoli A, Puccetti L, Bruni F, et al. Diastolic filling in hypertrophied hearts of elite runners: an echo-Doppler study. European review for medical and pharmacological sciences 2001;5(2):65-9.

164. Palazzuoli A, Puccetti L, Pastorelli M, et al. Transmitral and pulmonary venous flow study in elite male runners and young adults. International journal of cardiology 2002;84(1):47-51.

165. Pavlik G, Kemeny D, Kneffel Z, et al. Echocardiographic data in hungarian top-level water polo players. Medicine and science in sports and exercise 2005;37(2):323-8.

166. Pelliccia A, Adami PE, Quattrini F, et al. Are Olympic athletes free from cardiovascular diseases? Systematic investigation in 2352 participants from Athens 2004 to Sochi 2014. British journal of sports medicine 2017;51(4):238-43.

167. Pelliccia A, Culasso F, Di Paolo FM, et al. Physiologic left ventricular cavity dilatation in elite athletes. Annals of internal medicine 1999;130(1):23-31.

168. Pelliccia A, De Martino L, Borrazzo C, et al. Clinical correlates and outcome of the patterns of premature ventricular beats in Olympic athletes: a long-term follow-up study. European journal of preventive cardiology 2020:2047487320928452.

169. Pelliccia A, Di Paolo FM, De Blasiis E, et al. Prevalence and clinical significance of aortic root dilation in highly trained competitive athletes. Circulation 2010;122(7):698-706.

170. Pelliccia A, Kinoshita N, Pisicchio C, et al. Long-term clinical consequences of intense, uninterrupted endurance training in olympic athletes. Journal of the American College of Cardiology 2010;55(15):1619-25.

171. Pelliccia A, Maron BJ, Culasso F, et al. Clinical significance of abnormal electrocardiographic patterns in trained athletes. Circulation 2000;102(3):278-84.

172. Pelliccia A, Maron BJ, Culasso F, et al. Athlete's heart in women. Echocardiographic characterization of highly trained elite female athletes. JAMA 1996;276(3):211-5.

173. Perrin T, Trachsel LD, Schneiter S, et al. Prevalence of abnormal electrocardiograms in Swiss elite athletes detected with modern screening criteria. Swiss medical weekly 2016;146:w14376.

174. Perseghin G, Lattuada G, Terruzzi I, et al. Effect of the sporting discipline on the right and left ventricular morphology and function of elite male track runners: A magnetic resonance imaging and phosphorus 31 spectroscopy study. 2007;154(5):937-42.

175. Petersen SE, Hudsmith LE, Robson MD, et al. Sex-specific characteristics of cardiac function, geometry, and mass in young adult elite athletes. Journal of magnetic resonance imaging : JMRI 2006;24(2):297-303.

176. Poh K-K, Ton-Nu T-T, Neilan TG, et al. Myocardial adaptation and efficiency in response to intensive physical training in elite speedskaters. International journal of cardiology 2008;126(3):346-51.

177. Radonic V, Kozmar D, Pocanic D, et al. Mortality and causes of death among Croatian male Olympic medalists. Croatian medical journal 2017;58(4):263-69.

178. Real JT, Merchante A, Gomez JL, et al. Effects of marathon running on plasma total homocysteine concentrations. Nutrition, metabolism, and cardiovascular diseases : NMCD 2005;15(2):134-9.

179. Riley-Hagan M, Peshock RM, Stray-Gundersen J, et al. Left ventricular dimensions and mass using magnetic resonance imaging in female endurance athletes. 1992;69(12):1067-74.

180. Rimensberger C, Carlen F, Brugger N, et al. Right ventricular adaptations and arrhythmias in amateur ultra-endurance athletes. British journal of sports medicine 2014;48(15):1179-84.

181. Roberts WO, Roberts DM, Lunos S. Marathon related cardiac arrest risk differences in men and women. 2013;47(3):168-71.

182. Roberts WO, Schwartz RS, Garberich RF, et al. Fifty Men, 3510 Marathons, Cardiac Risk Factors, and Coronary Artery Calcium Scores. Medicine and science in sports and exercise 2017;49(12):2369-73.

183. Rocker L, Drygas WK, Heyduck B. Blood platelet activation and increase in thrombin activity following a marathon race. European journal of applied physiology and occupational physiology 1986;55(4):374-80.

184. Rodriguez-Plaza LG, Alfieri AB, Cubeddu LX. Urinary excretion of nitric oxide metabolites in runners, sedentary individuals and patients with coronary artery disease: effects of 42 km marathon, 15 km race and a cardiac rehabilitation program. Journal of cardiovascular risk 1997;4(5-6):367-72.

185. Rosin B. Is marathon running toxic? An observational study of cardiovascular disease prevalence and longevity in 54 male marathon runners. The Physician and sportsmedicine 2017;45(2):105-09.

186. Saenz AJ, Lee-Lewandrowski E, Lewandrowski KB, et al. Measurement of a plasma stroke biomarker panel and cardiac troponin T in marathon runners before and after the 2005 Boston Marathon. 2006;126(2):185-89.

187. Sanz-de la Garza M, Grazioli G, Bijnens BH, et al. Acute, Exercise Dose-Dependent Impairment in Atrial Performance During an Endurance Race: 2D Ultrasound Speckle-Tracking Strain Analysis. JACC Cardiovascular imaging 2016;9(12):1380-88.

188. Sarna S, Kaprio J, Kujala UM, et al. Health status of former elite athletes. The Finnish experience. Aging (Milan, Italy) 1997;9(1-2):35-41.

189. Sarna S, Sahi T, Koskenvuo M, et al. Increased life expectancy of world class male athletes. Medicine and science in sports and exercise 1993;25(2):237-44.

190. Scharf M, Uder M, Lell MM, et al. Atrial and ventricular functional and structural adaptations of the heart in elite triathletes assessed with cardiac MR imaging. 2010;257(1):71-79.

191. Scharhag J, Herrmann M, Urhausen A, et al. Independent elevations of N-terminal pro-brain natriuretic peptide and cardiac troponins in endurance athletes after prolonged strenuous exercise. American heart journal 2005;150(6):1128-34.

192. Scherr C, Fabiano LCC, Guerra RL, et al. Sports Practices and Cardiovascular Risk in Teenagers. Arquivos brasileiros de cardiologia 2018;110(3):248-55.

193. Scherr C, Helal L, Ferrari F, et al. The Olympic Experimental Gymnasium Program and its Association with the Prevalence of Cardiovascular Risk Factors in Adolescents: A Cross-Sectional Study. Arquivos brasileiros de cardiologia 2019;112(6):775-81.

194. Scherr J, Schuster T, Pressler A, et al. Repolarization perturbation and hypomagnesemia after extreme exercise. Medicine and science in sports and exercise 2012;44(9):1637-43.

195. Shave RE, Dawson E, Whyte G, et al. Evidence of exercise-induced cardiac dysfunction and elevated cTnT in separate cohorts competing in an ultra-endurance mountain marathon race. International journal of sports medicine 2002;23(7):489-94.

196. Siegel AJ, Lewandrowski EL, Chun KY, et al. Changes in cardiac markers including B-natriuretic peptide in runners after the Boston marathon. 2001;88(8):920-23.

197. Siegel AJ, Lewandrowski KB, Strauss HW, et al. Normal post-race antimyosin myocardial scintigraphy in asymptomatic marathon runners with elevated serum creatine kinase MB isoenzyme and troponin T levels. Evidence against silent myocardial cell necrosis. Cardiology 1995;86(6):451-6.

198. Siegel AJ, Silverman LM, Evans WJ. Elevated skeletal muscle creatine kinase MB isoenzyme levels in marathon runners. JAMA 1983;250(20):2835-7.

199. Siegel AJ, Silverman LM, Holman BL. Elevated creatine kinase MB isoenzyme levels in marathon runners. Normal myocardial scintigrams suggest noncardiac source. JAMA 1981;246(18):2049-51.

200. Siegel AJ, Silverman LM, Holman BL. Normal results of post-race thallium-201 myocardial perfusion imaging in marathon runners with elevated serum MB creatine kinase levels. The American journal of medicine 1985;79(4):431-4.

201. Simsek Z, Hakan Tas M, Degirmenci H, et al. Speckle tracking echocardiographic analysis of left ventricular systolic and diastolic functions of young elite athletes with eccentric and concentric type of cardiac remodeling. Echocardiography (Mount Kisco, NY) 2013;30(10):1202-8.

202. Sivertssen E, Benestad AM, Rykke E, et al. [Is top level athletic performance dangerous? A 25-year follow-up study of 24 elite cross-country skiers]. Er det farlig a drive toppidrett? En 25 ars etterundersokelse av 24 elitelangrennslopere 1994;114(28):3300-4.

203. Skadberg O, Kleiven O, Bjorkavoll-Bergseth M, et al. Highly increased Troponin I levels following high-intensity endurance cycling may detect subclinical coronary artery disease in presumably healthy leisure sport cyclists: The North Sea Race Endurance Exercise Study (NEEDED) 2013. European journal of preventive cardiology 2017;24(8):885-94.

204. Smith GS, Vacek JL, Wilson DB, et al. Exercise-induced alterations of signal-averaged electrocardiograms in marathon runners. American heart journal 1989;118(6):1198-202.

205. Smith WG, Cullen KJ, Thorburn IO. ELECTROCARDIOGRAMS OF MARATHON RUNNERS IN 1962 COMMONWEALTH GAMES. British heart journal 1964;26:469-76.

206. Spirito P, Pelliccia A, Proschan MA, et al. Morphology of the "athlete's heart" assessed by echocardiography in 947 elite athletes representing 27 sports. The American journal of cardiology 1994;74(8):802-6.

207. Starekova J, Thottakara T, Lund GK, et al. Increased myocardial mass and attenuation of myocardial strain in professional male soccer players and competitive male triathletes. The international journal of cardiovascular imaging 2020

208. Stoggl T, Schwarzl C, Muller EE, et al. Effects of skiing on cardiorespiratory and metabolic responses in middle-aged subjects with increased cardiovascular risk. International journal of cardiology 2016;203:618-20.

209. Stoutenberg M, Kressler J, Chen GL, et al. Aerobic training does not alter CRP in apparently healthy, untrained men. The Journal of sports medicine and physical fitness 2012;52(1):53-62.

210. Stray-Gundersen J, Denke MA, Grundy SM. Influence of lifetime cross-country skiing on plasma lipids and lipoproteins. Medicine and science in sports and exercise 1991;23(6):695-702.

211. Svedberg N, Sundstrom J, James S, et al. Long-Term Incidence of Atrial Fibrillation and Stroke Among Cross-Country Skiers. 2019;140(11):910-20.

212. Swiatowiec A, Krol W, Kuch M, et al. Analysis of 12-lead electrocardiogram in top competitive professional athletes in the light of recent guidelines. Kardiologia polska 2009;67(10):1095-102.

213. Swift DL, Church TS, Earnest CP, et al. The effect of different doses of aerobic exercise training on exercise blood pressure in overweight and obese postmenopausal women. 2012;19(5):503-09.

214. Swift DL, Johannsen NM, Earnest CP, et al. Effect of different doses of aerobic exercise training on total bilirubin levels. Medicine and science in sports and exercise 2012;44(4):569-74.

215. Swift DL, Johannsen NM, Lavie CJ, et al. Racial differences in the response of cardiorespiratory fitness to aerobic exercise training in Caucasian and African American postmenopausal women. Journal of applied physiology (Bethesda, Md : 1985) 2013;114(10):1375-82.

216. Szauder I, Kovacs A, Pavlik G. Comparison of left ventricular mechanics in runners versus bodybuilders using speckle tracking echocardiography. Cardiovascular ultrasound 2015;13:7.

217. Tahir E, Scherz B, Starekova J, et al. Acute impact of an endurance race on cardiac function and biomarkers of myocardial injury in triathletes with and without myocardial fibrosis. European journal of preventive cardiology 2020;27(1):94-104.

218. Tahir E, Starekova J, Muellerleile K, et al. Myocardial Fibrosis in Competitive Triathletes Detected by Contrast-Enhanced CMR Correlates With Exercise-Induced Hypertension and Competition History. JACC Cardiovascular imaging 2018;11(9):1260-70.

219. Tian Y, Nie J, Tong TK, et al. Changes in serum cardiac troponins following a 21-km run in junior male runners. The Journal of sports medicine and physical fitness 2006;46(3):481-8.

220. Trivax JE, Franklin BA, Goldstein JA, et al. Acute cardiac effects of marathon running. Journal of applied physiology (Bethesda, Md : 1985) 2010;108(5):1148-53.

221. Trullas JC, Roca E, Guillermo A, et al. Ultra Pirineu 2017: caracteristicas de los corredores de elite y de no elite y efectos sobre la salud de una maraton de montana: estudio piloto Serialmed-UPUltra Pirineu 2017: Characteristics of elite and non-elite runners and effects on health of a mountain marathon: Serialmed-UP pilot study. 2018;53(200):139-46.

222. Tsiflikas I, Thomas C, Fallmann C, et al. Prevalence of Subclinical Coronary Artery Disease in Middle-Aged, Male Marathon Runners Detected by Cardiac CT. RoFo : Fortschritte auf dem Gebiete der Rontgenstrahlen und der Nuklearmedizin 2015;187(7):561-8.

223. Ujka K, Bastiani L, D'Angelo G, et al. Enhanced Right-Chamber Remodeling in Endurance Ultra-Trail Athletes Compared to Marathon Runners Detected by Standard and Speckle-Tracking Echocardiography. Frontiers in physiology 2017;8:527.

224. van Buuren F, Mellwig KP, Butz T, et al. Left ventricular mass and oxygen uptake in top handball athletes. International journal of sports medicine 2013;34(3):200-6.

225. Van Buuren F, Mellwig KP, Faber L, et al. The occurrence of atrial fibrillation in former top-level handball players above the age of 50. Acta cardiologica 2012;67(2):213-20.

226. Vance DD, Chen GL, Stoutenberg M, et al. Cardiac performance, biomarkers and gene expression studies in previously sedentary men participating in half-marathon training. BMC sports science, medicine & rehabilitation 2014;6(1):6.

227. Vassalle C, Masotti S, Lubrano V, et al. Traditional and new candidate cardiac biomarkers assessed before, early, and late after half marathon in trained subjects. European journal of applied physiology 2018;118(2):411-17.

228. Vicent L, Ariza-Sole A, Gonzalez-Juanatey JR, et al. Exercise-related severe cardiac events. Scandinavian journal of medicine & science in sports 2018;28(4):1404-11.

229. Vidotto C, Atamaniuk J, Muller MM, et al. Responses of N-terminal pro-brain natriuretic peptide (NT-proBNP) and cardiac troponin I (cTnI) to competitive endurance exercise in recreational athletes. 2005;26(8):645-50.

230. Virmani R. Jogging, marathon running, and death. 1982;8(4):96-107.

231. Vitarelli A, Capotosto L, Placanica G, et al. Comprehensive assessment of biventricular function and aortic stiffness in athletes with different forms of training by three-dimensional echocardiography and strain imaging. European heart journal cardiovascular Imaging 2013;14(10):1010-20.

232. Warburton DER, Welsh RC, Haykowsky MJ, et al. Effects of half ironman competition on the development of late potentials. Medicine and science in sports and exercise 2000;32(7):1208-13.

233. Whyte G, George K, Stephenson C, et al. Impact of marathon running on cardiac structure and function in recreational runners. 2005;108(1):73-80.

234. Whyte G, Sharma S, McKenna WJ, et al. Alterations in cardiac morphology and function in elite multi-disciplinary athletes. 1999;20(4):222-26.

235. Whyte GP, George K, Sharma S, et al. The upper limit of physiological cardiac hypertrophy in elite male and female athletes: the British experience. European journal of applied physiology 2004;92(4-5):592-7.

236. Whyte GP, Sharma S, George K, et al. Exercise gas exchange responses in the differentiation of pathologic and physiologic left ventricular hypertrophy. Medicine and science in sports and exercise 1999;31(9):1237-41.

237. Wilhelm M, Nuoffer J-M, Schmid J-P, et al. Comparison of pro-atrial natriuretic peptide and atrial remodeling in marathon versus non-marathon runners. The American journal of cardiology 2012;109(7):1060-5.

238. Wilhelm M, Roten L, Tanner H, et al. Long-term cardiac remodeling and arrhythmias in nonelite marathon runners. The American journal of cardiology 2012;110(1):129-35.

239. Wilhelm M, Roten L, Tanner H, et al. Atrial remodeling, autonomic tone, and lifetime training hours in nonelite athletes. The American journal of cardiology 2011;108(4):580-5.

240. Wilhelm M, Roten L, Tanner H, et al. Gender differences of atrial and ventricular remodeling and autonomic tone in nonelite athletes. The American journal of cardiology 2011;108(10):1489-95.

241. Wilhelm M, Zueger T, De Marchi S, et al. Inflammation and atrial remodeling after a mountain marathon. Scandinavian journal of medicine & science in sports 2014;24(3):519-25.

242. Williams PT. High-density lipoprotein cholesterol and other risk factors for coronary heart disease in female runners. The New England journal of medicine 1996;334(20):1298-303.

243. Williams PT. Relationship of distance run per week to coronary heart disease risk factors in 8283 male runners. The National Runners' Health Study. Archives of internal medicine 1997;157(2):191-8.

244. Williams PT, Franklin BA. Reduced incidence of cardiac arrhythmias in walkers and runners. PloS one 2013;8(6):e65302.

245. Yu HH, Ginsburg GS, O'Toole ML, et al. Acute changes in serum lipids and lipoprotein subclasses in triathletes as assessed by proton nuclear magnetic resonance spectroscopy. Arteriosclerosis, thrombosis, and vascular biology 1999;19(8):1945-9.

246. Zebrowska A, Mikolajczyk R, Waskiewicz Z, et al. Acute responses of novel cardiac biomarkers to a 24-h ultra-marathon. 2019;8(1)

247. Zha W, Schiros CG, Reddy G, et al. Improved Right Ventricular Performance with Increased Tricuspid Annular Excursion in Athlete's Heart. Frontiers in cardiovascular medicine 2015;2:8.

248. Zilinski JL, Contursi ME, Isaacs SK, et al. Myocardial adaptations to recreational marathon training among middle-aged men. Circulation Cardiovascular imaging 2015;8(2):e002487.

249. Ziman MR, Jeenah MS. The relation between the PST1 restriction fragment length polymorphism at the APO-AI locus and plasma APO-AI concentrations in marathon runners. South African medical journal = Suid-Afrikaanse tydskrif vir geneeskunde 1995;85(10):1013-6.

250. Zorzi A, Mastella G, Cipriani A, et al. Burden of ventricular arrhythmias at 12-lead 24-hour ambulatory ECG monitoring in middle-aged endurance athletes versus sedentary controls. European journal of preventive cardiology 2018;25(18):2003-11.

Supplement 5 – Table showing characteristics of the studies included within the scoping review

Study Number	Harvard Reference	Number of participants	Number of females	Percentage female	Location	Exercise Type
1	Starekova et al. 2020	58	0	0.0%	European	Other
2	Mango et al. 2020	516	204	39.5%	European	Mixed
3	Pelliccia et al 2020	4595	1610	35.0%	European	Mixed
4	D'Silva et al 2020	68	32	47.1%	European	Running
5	Bjerring et al. 2020	36	12	33.3%	European	Cross-Country Skiing
6	Lewicka-Potocka et al. 2020	49	0	0.0%	European	Running
7	Bernardino et al. 2020	166	74	44.6%	European	Triathlon
8	Tahir et al. 2020	50	0	0.0%	European	Triathlon
9	D'Silva et al 2020	120	60	50.0%	European	Running
10	Miranda et al. 2020	88	26	29.5%	Americas	Other
11	Kunutsor et al. 2020	1949	0	0.0%	European	Cross-Country Skiing
12	Hosatte-Ducassy et al. 2020	99	41	41.4%	Americas	Triathlon
13	Dawkins et al. 2019	66	13	19.7%	European	Triathlon

14	Bjerring et al. 2019	48	14	29.2%	European	Cross-Country Skiing
15	Kunutsor et al 2019	1809	0	0.0%	European	Cross-Country Skiing
16	Jin et al 2019	501690	250664	50.0%	Western Pacific	Other
17	Major et al. 2019	363	109	30.0%	European	Mixed
18	Edwards and Loprinzi 2019	6694	3481	52.0%	Americas	Other
19	Hochsmann et al. 2019	137	97	70.8%	Americas	Other
20	Scheer et al 2019	1113	606	54.4%	Americas	Mixed
21	Malek et al 2019	40	0	0.0%	European	Running
22	Leckie et ak 2019	165	62	37.6%	European	Running
23	Hirata et al. 2019	12	3	25.0%	Western Pacific	Running
24	D'Ascenzi et al. 2019	1058	402	38.0%	European	Mixed
25	Zebrowska et al. 2019	14	0	0.0%	European	Running
26	Svedberg et al. 2019	736102	256378	34.8%	European	Cross-Country Skiing
27	Zorzi et al 2018	270	41	15.2%	European	Mixed
28	Gabrielli et al. 2018	16	0	0.0%	Americas	Running
29	Kaleta et al. 2018	40	0	0.0%	European	Running

30	Bjerring et al. 2018	101	41	40.6%	European	Cross-Country Skiing
31	Opondo et al. 2018	61	35	57.4%	Americas	Other
32	Antero-jacquemin et al. 2018	2814	696	24.7%	European	Mixed
33	Krumm et al. 2018	18	3	16.7%	European	Mixed
34	Scherr et al 2018	422	217	51.4%	Americas	Mixed
35	Vassalle et al. 2018	18	3	16.7%	European	Running
36	Tahir et al. 2018	119	43	36.1%	European	Triathlon
37	Vicent et al. 2018	117	8	6.8%	European	Mixed
38	Trullas et al. 2018	25	4	16.0%	European	Running
39	Harris et al. 2017	135	20	14.8%	Americas	Triathlon
40	Radonic et al. 2017	233	0	0.0%	European	Mixed
41	Herm et al. 2017	109	26	23.9%	European	Running
42	Roberts et al. 2017	50	0	0.0%	Americas	Running
43	Aengevaeren et al. 2017	284	0	0.0%	European	Mixed
44	Skadberg et al. 2017	97	23	23.7%	European	Mixed
45	Rosin 2017	54	0	0.0%	Americas	Running

46	Pelliccia et al. 2017	2352	919	39.1%	European	Mixed
47	Bonsignore et al. 2017	71	9	12.7%	Americas	Running
48	Dupont et al. 2017	24	0	0.0%	European	Triathlon
49	D'Ascenzi et al. 2017	1009	362	35.9%	European	Mixed
50	Ujka et al. 2017	92	22	23.9%	European	Running
51	Merghani et al. 2017	244	84	34.4%	European	Running
52	Perrin et al. 2016	287	103	35.9%	European	Mixed
53	Abdullah et al. 2016	92	25	27.2%	Americas	Other
54	Sanz de la Garza et al. 2016	60	0	0.0%	European	Running
55	Clauss et al. 2016	30	0	0.0%	European	Running
56	Stoggl et al. 2016	21	8	38.1%	European	Cross-Country Skiing
57	Calvo et al. 2016	172	22	12.8%	European	Other
58	Myrstad et al. 2016	4952	318	6.4%	European	Cross-Country Skiing
59	Konopka et al. 2016	117	53	45.3%	European	Other
60	Lemez et al. 2016	205	0	0.0%	Americas	Mixed
61	Hallmarker et al. 2016	204038	77534	38.0%	European	Cross-Country Skiing

62	Martin-Beaulieu et al. 2016	22	13	59.1%	Americas	Running
63	Montiel et al. 2016	18	18	100.0%	European	Running
64	Zha et al. 2015	60	30	50.0%	Americas	Running
65	Lacey et al. 2015	12203	0	0.0%	Western Pacific	Other
66	Loprinzi et al. 2015	16049	8121	50.6%	Americas	Other
67	Grabs et al. 2015	20	0	0.0%	European	Running
68	Szauder et al. 2015	53	0	0.0%	European	Running
69	Arem et al. 2015	661137	369652	55.9%	Americas	Other
70	Tsifilikas et al. 2015	50	0	0.0%	European	Running
71	Antero-Jacquemin et al. 2015	2812	726	25.8%	European	Mixed
72	Davis et al. 2015	71	33	46.5%	Americas	Other
73	Zilinski et al. 2015	45	0	0.0%	Americas	Running
74	Gram et al. 2015	61	0	0.0%	European	Other
75	Demirelli et al. 2015	73	0	0.0%	European	Running
76	Caselli et al. 2015	1299	526	40.5%	European	Mixed
77	McClean et al. 2015	56	0	0.0%	European	Mixed

78	Lord et al. 2015	15	1	6.7%	Americas	Running
79	Kettunen et al. 2015	4387	0	0.0%	European	Mixed
80	Jouffroy et al. 2015	66	0	0.0%	European	Running
81	Caselli et al. 2015	200	79	39.5%	European	Mixed
82	Vance et al. 2014	47	0	0.0%	Americas	Running
83	Antero-Jacquemin et al. 2014	255	0	0.0%	European	Other
84	Myrstad et al. 2014	6358	0	0.0%	European	Cross-Country Skiing
85	Franco et al. 2014	19	5	26.3%	Americas	Running
86	Rimensberger et al. 2014	105	0	0.0%	European	Running
87	Myrstad et al. 2014	3364	0	0.0%	European	Cross-Country Skiing
88	Mohlenkamp et al. 2014	1188	0	0.0%	European	Running
89	Hattasch et al. 2014	22	0	0.0%	European	Running
90	Wilhelm et al. 2014	11	0	0.0%	European	Running
91	Leischik et al. 2014	51	0	0.0%	European	Triathlon
92	Leischik and Spelsberg 2014	87	33	37.9%	European	Triathlon
93	Kim et al. 2014	249	0	0.0%	Western Pacific	Running

94	Dalla Vecchia etal. 2014	35	4	11.4%	Americas	Running
95	Marijon et al. 2013	786	0	0.0%	European	Cross-Country Skiing
96	Denham et al. 2013	130	0	0.0%	European	Running
97	Gaudreault et al. 2013	20	14	70.0%	Americas	Running
98	Simsek et al. 2013	77	0	0.0%	European	Mixed
99	Williams and Franklin 2013	46907	27854	59.4%	European	Mixed
100	Andersen et al. 2013	54560	5443	10.0%	European	Cross-Country Skiing
101	Kim et al. 2013	59	0	0.0%	Western Pacific	Running
102	Swift et al. 2013	464	464	100.0%	Americas	Other
103	Karstoft et al. 2013	8	1	12.5%	European	Running
104	Vitarelli et al. 2013	140	0	0.0%	European	Mixed
105	Franzen et al. 2013	40	0	0.0%	European	Mixed
106	Van Buuren et al. 2013	291	0	0.0%	European	Other
107	Iellamo et al. 2013	20	0	0.0%	European	Other
108	Iellamo et al. 2013	20	0	0.0%	European	Other
109	Roberts et al. 2013	548092	168227	30.7%	Americas	Running

110	Haeusler et al. 2012	110	27	24.5%	European	Running
111	Kalstedt et al. 2012	25	4	16.0%	Americas	Running
112	Van Buuren et al. 2012	33	0	0.0%	European	Other
113	Mathews et al. 2012	3718336	1463276	39.4%	Americas	Running
114	Swift et al. 2012	464	464	100.0%	Americas	Other
115	Scherr et al. 2012	277	0	0.0%	European	Running
116	Wilhelm et al. 2012	138	68	49.3%	European	Running
117	Stoutenberg Et Al 2012	42	0	0.0%	Americas	Running
118	Kim et al. 2012	10871000	4862670	44.7%	Americas	Running
119	Wilhelm et al. 2012	70	0	0.0%	European	Running
120	Caselli et al. 2012	92	28	30.4%	European	Other
121	Swift Et Al 2012	94	94	100.0%	Americas	Other
122	Conti et al. 2012	35	0	0.0%	European	Mixed
123	Wilhelm et al. 2011	138	68	49.3%	European	Running
124	Wilhelm et al. 2011	70	0	0.0%	European	Running
125	Krol et al. 2011	79	14	17.7%	European	Mixed

126	Grimsmo et al. 2011	122	0	0.0%	European	Cross-Country Skiing
127	Durand et al. 2011	527	0	0.0%	Americas	Other
128	Minns et al. 2011	87	41	47.1%	Americas	Running
129	Hanssen et al. 2011	30	0	0.0%	European	Running
130	Pelliccia et al. 2010	2317	1017	43.9%	European	Mixed
131	Banfi et al. 2010	15	2	13.3%	European	Running
132	Pelliccia et al. 2010	114	25	21.9%	European	Mixed
133	Trivax et al. 2010	25	13	52.0%	Americas	Running
134	Grimsmo et al. 2010	149	0	0.0%	Americas	Cross-Country Skiing
135	Andersson et al. 2010	20	0	0.0%	European	Cross-Country Skiing
136	O'Hanlon et al. 2010	17	0	0.0%	European	Running
137	Scharf et al. 2010	26	0	0.0%	European	Triathlon
138	Indermuhle et al. 2009	60	4	6.7%	European	Triathlon
139	Swiatowiec et al. 2009	73	20	27.4%	European	Mixed
140	Her et al. 2009	75	25	33.3%	Western Pacific	Running
141	Hawkins, 2009	16	0	0.0%	Americas	Mixed

142	Nassenstein, 2009	108	0	0.0%	European	Running
143	Breuckmann et al. 2009	102	0	0.0%	European	Running
144	Nottin et al. 2009	23	0	0.0%	European	Triathlon
145	Heinz et al 2009	80	41	51.3%	European	Mixed
146	Lippi, 2008	17	0	0.0%	European	Running
147	Lippi et al 2008	17	0	0.0%	European	Running
148	Molina L, 2008	557	0	0.0%	European	Running
149	Kasikcioglu et al 2008	13	0	0.0%	European	Running
150	Poh, 2008	39	16	41.0%	Americas	Other
151	Koller et al. 2008	59	7	11.9%	European	Running
152	Lalande, 2007	19	0	0.0%	Western Pacific	Other
153	Belonje, 2007	62862	47775	76.0%	European	Mixed
154	Carlsson M, 2007	36	13	36.1%	European	Triathlon
155	Musat et al. 2007	30	30	100.0%	European	Running
156	Perseghin et al. 2007	33	0	0.0%	European	Running
157	Lippi, 2006	202	0	0.0%	European	Mixed

158	Tian Y, 2006	10	0	0.0%	Western Pacific	Running
159	Petersen et al 2006	81	37	45.7%	European	Mixed
160	Indermuhle A, 2006	93	60	64.5%	European	Mixed
161	Middleton , 2006	14	1	7.1%	European	Running
162	Saenz et al. 2006	35	6	17.1%	Americas	Running
163	Banfi, 2005	49	4	8.2%	European	Triathlon
164	Scharhag, 2005	105	13	12.4%	European	Running
165	Arrese, 2005	188	54	28.7%	European	Running
166	Real et al 2005	22	0	0.0%	European	Running
167	Makan, 2005	1150	309	26.9%	European	Running
168	Pavlik, 2005	65	0	0.0%	European	Mixed
169	Whyte et al. 2005	52	9	17.3%	European	Running
170	Vidotto et al. 2005	25	13	52.0%	European	Running
171	Whyte, 2004	442	136	30.8%	European	Mixed
172	Bennekers et al 2004	9	2	22.2%	European	Running
173	Date, 2003	51	0	0.0%	Western Pacific	Mixed

174	Galetta, 2003	48	0	0.0%	European	Mixed
175	Iwasaki, 2003	11	5	45.5%	Americas	Other
176	Herrmann et al. 2003	100	13	13.0%	European	Mixed
177	Draheim et al. 2003	145	72	49.7%	European	Mixed
178	Shave et al. 2002	37	0	0.0%	European	Running
179	Palazzuoli, 2002	45	0	0.0%	European	Running
180	Apple, 2002	19	12	63.2%	Americas	Running
181	Palazzuoli, 2001	43	0	0.0%	European	Running
182	Siegel, 2001	82	0	0.0%	Americas	Running
183	Matsuo et al 2001	48	10	20.8%	Western Pacific	Mixed
184	Ohba, 2001	13	0	0.0%	Western Pacific	Running
185	Claessens et al. 2001	74	0	0.0%	European	Triathlon
186	Ginsburg et al. 2001	57	19	33.3%	Americas	Triathlon
187	Claessens, 2000	74	0	0.0%	European	Triathlon
188	Dressendorfer, 2000	21	0	0.0%	Americas	Running
189	Hildick-Smith, 2000	52	0	0.0%	European	Mixed

190	Warburton et al. 2000	9	0	0.0%	Americas	Triathlon
191	Pelliccia, 2000	1005	260	25.9%	European	Mixed
192	Mattusch et al. 2000	14	0	0.0%	European	Running
193	Claessens et al. 2000	74	0	0.0%	European	Triathlon
194	Claessens, 1999	74	0	0.0%	European	Triathlon
195	Claessens, 1999	74	0	0.0%	European	Triathlon
196	Lucia et al. 1999	10	1	10.0%	European	Running
197	Whyte,1999	30	0	0.0%	European	Triathlon
198	Yu et al. 1999	28	6	21.4%	Americas	Triathlon
199	Damm et al. 1999	95	0	0.0%	European	Mixed
200	Mayet, 1999	33	0	0.0%	European	Mixed
201	Pelliccia, 1999	1309	352	26.9%	European	Mixed
202	Biffi et al. 1999	153	0	0.0%	European	Mixed
203	Whyte et al. 1999	29	0	0.0%	European	Mixed
204	Furlanello, 1998	1772	308	17.4%	European	Mixed
205	Rodriguez-Plaza	50	0	0.0%	Americas	Running

206	Bergstrom, 1997	879	402	45.7%	European	Running
207	Sarna, 1997	1712	0	0.0%	European	Mixed
208	Williams, 1997	8283	0	0.0%	Americas	Running
209	Cubeddu et al. 1997	50	0	0.0%	Americas	Running
210	Henriksen, 1996	127	0	0.0%	European	Mixed
211	Ketelhut, 1996	30	0	0.0%	European	Running
212	Ginsburg, 1996	39	13	33.3%	Americas	Triathlon
213	Pelliccia, 1996	665	665	100.0%	European	Mixed
214	Williams, 1996	1837	1837	100.0%	Americas	Running
215	Biffi et al. 1996	60	0	0.0%	European	Mixed
216	Ziman and Jeenah 1995	82	0	0.0%	Africa	Running
217	Siegel, 1995	8	0	0.0%	Americas	Running
218	Kokkinos et al. 1995	2906	0	0.0%	Americas	Running
219	Maron, 1994	14	1	7.1%	Americas	Mixed
220	Luurila, 1994	37	0	0.0%	European	Cross-Country Skiing
221	Spirito, 1994	947	209	22.1%	European	Mixed

222	Martinez-Mas, 1994	42	0	0.0%	European	Running
223	Imai, 1994	60	0	0.0%	Western Pacific	Cross-Country Skiing
224	Sivertssen, 1994	24	11	45.8%	European	Cross-Country Skiing
225	Sarna, 1993	2613	0	0.0%	European	Mixed
226	Maron, 1993	6	0	0.0%	European	Mixed
227	Riley-Hagan et al. 1992	46	46	100.0%	Americas	Mixed
228	Ogawa, 1991	54	0	0.0%	Western Pacific	Cross-Country Skiing
229	Stray-Gundersen, 1991	176	48	27.3%	Americas	Cross-Country Skiing
230	Morris, 1990	9376	0	0.0%	European	Other
231	Kostka, 1989	194	0	0.0%	European	Running
232	Smith, 1989	30	3	10.0%	Americas	Running
233	Rocker, 1986	16	0	0.0%	European	Running
234	Siegel, 1985	15	0	0.0%	Americas	Running
235	Lie, 1985	2136	0	0.0%	European	Cross-Country Skiing
236	Paffenbarger, 1984	572	0	0.0%	Americas	Other
237	Apple, 1984	12	0	0.0%	Americas	Running

238	Lie, 1984	122	0	0.0%	European	Cross-Country Skiing
239	Apple, 1983	2	0	0.0%	Americas	Running
240	Siegel, 1983	35	0	0.0%	Americas	Running
241	Deshaies, 1982	95	31	32.6%	Americas	Mixed
242	Ohman, 1982	15	0	0.0%	European	Running
243	Virmani et al. 1982	30	0	0.0%	Americas	Running
244	Siegel,1981	35	0	0.0%	Americas	Running
245	Murayama and Kuroda 1980	365	64	17.5%	Western Pacific	Triathlon
246	Olivier, 1978	48	0	0.0%	Africa	Running
247	Lie, 1978	149	0	0.0%	European	Cross-Country Skiing
248	Milvy, 1977	9958	0	0.0%	Americas	Running
249	Nakamoto, 1969	72	27	37.5%	Western Pacific	Running
250	Smith, 1964	21	0	0.0%	Western Pacific	Mixed
